# Focal adhesion in the tumour metastasis: from molecular mechanisms to therapeutic targets

**DOI:** 10.1186/s40364-025-00745-7

**Published:** 2025-03-05

**Authors:** Zonghao Liu, Xiaofang Zhang, Tianru Ben, Mo Li, Yi Jin, Tianlu Wang, Yingqiu Song

**Affiliations:** 1 Department of Radiotherapy, Cancer Hospital of China Medical University, No.44 Xiaoheyan Road, Dadong District, Shenyang, Liaoning Province 110042 P. R. China; 2 The First Clinical College, China Medical University, Shenyang, Liaoning Province 110122 P. R. China; 3 Department of Medical Oncology, The First Hospital of China Medical University, Shenyang, Liaoning 110001 China; 4 Department of Breast Surgery, Liaoning Cancer Hospital and Institute, No.44 Xiaoheyan Road, Dadong District, Shenyang, Liaoning Province 110042 P. R. China; 5 Department of Radiotherapy, Cancer Hospital of Dalian University of Technology, Shenyang, Liaoning Province 110042 People’s Republic of China; 6 Faculty of Medicine, Dalian University of Technology, Dalian, Liaoning Province 116024 P. R. China; 7 Department of Radiotherapy, Liaoning Cancer Hospital & Institute, No.44 Xiaoheyan Road, Dadong District, Shenyang, Liaoning Province 110042 P. R. China

## Abstract

The tumour microenvironment is the “hotbed” of tumour cells, providing abundant extracellular support for growth and metastasis. However, the tumour microenvironment is not static and is constantly remodelled by a variety of cellular components, including tumour cells, through mechanical, biological and chemical means to promote metastasis. Focal adhesion plays an important role in cell-extracellular matrix adhesion. An in-depth exploration of the role of focal adhesion in tumour metastasis, especially their contribution at the biomechanical level, is an important direction of current research. In this review, we first summarize the assembly of focal adhesions and explore their kinetics in tumour cells. Then, we describe in detail the role of focal adhesion in various stages of tumour metastasis, especially its key functions in cell migration, invasion, and matrix remodelling. Finally, we describe the anti-tumour strategies targeting focal adhesion and the current progress in the development of some inhibitors against focal adhesion proteins. In this paper, we summarize for the first time that focal adhesion play a positive feedback role in pro-tumour metastatic matrix remodelling by summarizing the five processes of focal adhesion assembly in a multidimensional way. It is beneficial for researchers to have a deeper understanding of the role of focal adhesion in the biological behaviour of tumour metastasis and the potential of focal adhesion as a therapeutic target, providing new ideas for the prevention and treatment of metastases.

## Introduction

Tumour metastasis is the leading cause of death in cancer patients, accounting for approximately 90% of all cancer deaths [1]. Despite significant advances in the early diagnosis and treatment of tumours, effective therapeutic strategies for metastasis are still limited. Tumour metastasis is a complex process, and does not occur by chance. The “seed and soil” theory proposed by Stephen Paget et al. has revealed that the secondary growth sites of metastatic tumours are the result of a number of contributing factors [2]. Jawad Fares et al. summarised the molecular mechanisms of tumour metastasis, involving the coordination of primary tumour sites and secondary growth sites with each other [3]. This process is poorly understood and therefore limited results have been achieved in the prevention and inhibition of tumour metastasis. Therefore, further understanding of the mechanisms of metastasis is essential to prevent and suppress tumour metastasis.

Cells, as the basic structural and functional units of organisms, continuously receive and sense extracellular signals (e.g. physical, chemical, and biological signals), and provide feedback to regulate cellular behaviour accordingly. Cellular “mechanotransduction” refers to the ability of cells to sense mechanical signals in the extracellular matrix (ECM) through various cellular structures and convert mechanical signals into biochemical signals, including the activation of signalling pathways and changes in the location/activity of associated proteins [4]. The main player in this process is focal adhesion. Focal adhesion is connected to the actin-associated cytoskeleton on the intracellular side and to the ECM on the extracellular side. It acts as a “mechanosensor" of the cell, sensing and transmitting mechanical signals inside and outside the cell [5]. This mechanism enables focal adhesion to communicate bi-directionally between the cell and its microenvironment, regulating cell behaviour and responding to external mechanical stimuli. Additionally, unlike other mechanosensor, focal adhesion is unique in that it not only senses mechanical signals, but also serves as an intersection of biochemical and mechanical signals through the interactions between integrins on it and intracellular biochemical signal receptors. Thus, focal adhesion serves as a key signalling hub capable of integrating multiple signals from inside and outside the cell, providing the basis for the regulation of cellular behaviour.

Numerous studies have shown that focal adhesion proteins play key roles in the deterioration and progression of a wide range of tumours. Yam et al. summarised the synergistic roles of several focal adhesion proteins such as integrin, integrin-linked kinase (ILK) and Grb7 in the development and metastasis of hepatocellular carcinoma (HCC) [6]. Meanwhile, these proteins have demonstrated significant clinical relevance. For example, a study based on 69 patients with HCC showed that ILK was overexpressed in all HCC samples and that this phenomenon was strongly associated with cirrhosis-associated activation of the Akt signalling pathway [7]. In another study of HCC samples (100 cases), *DLC1* expression was found to be significantly down-regulated, and its low expression may promote tumour invasion and metastasis by disrupting Rho-GTPase activity, thereby affecting the dynamic remodelling of the cytoskeleton [8]. In addition, integrins and focal adhesion proteins modulate treatment resistance in a variety of tumour cells, including glioma, breast cancer and uroepithelial carcinoma, through interactions with growth factor (GF) receptors such as epidermal growth factor receptor (EGFR) [9–11]. For example, blocking αvβ3 and αvβ5 significantly increases the sensitivity of glioma cells to ionising radiation and reduces the survival of irradiated radioresistant tumour cells after irradiation [9]. In a study of patients with uroepithelial carcinoma (72 cases), mutations in the integrin signalling pathway following chemotherapy were found to induce functionally acquired molecular alterations, which further promoted malignant tumour progression by enhancing cell survival and drug resistance [11]. Table 1 summarises the clinicopathological relevance of focal adhesion proteins, demonstrating the expression profile of these proteins and their clinical significance. Such examples further illustrate the important role of these proteins in tumour progression and drug resistance mechanisms.

**Table 1:** Expression and clinicopathological significance of focal adhesion proteins

Focal adhesion protein	Tumour types	Total number of samples	Expression	Clinical relevance	References
Integrin α11β1	Breast cancer	392	Differentially expressed in the vast majority (99%)	High expression (66%) correlates with invasive characteristics	[12]
Integrin α4	GIST	147	Expressed in 62 (42.2%)	Expression correlates with distant metastasis and poor prognostic features	[13]
Integrin β4	Breast cancer	105	Expression is restricted	Expression correlates with basal-like features of tumour cells and promotes tumour invasive behaviour	[14]
FAK	Ameloblastoma	44	Strongly expressed in 36 cases (82%), weakly expressed in 8 cases (18%)	-	[15]
FAK	OSCC	96	-	Significantly lower OS	[16]
FAK	Breast cancer	82	Twenty-one women (26%) had high expression and 61 (74%) had low expression	High expression was an independent poor prognostic factor; Low expression correlates with improved chemosensitivity and 5-year relapse-free survival	[17]
FAK	HCC	60	Overexpressed in HCC (*P* = 0.0008)	Overexpression was significantly associated with tumour size (*P* = 0.034) and serum AFP levels (*P* = 0.030)	[18]
FAK	HCC	64	Cytoplasmic expression was observed in 18 cases (28.1%)	Expression correlates with low serum albumin levels and portal vein infiltration	[19]
Src	Osteosarcoma	60	-	A correlation between Src subcellular localisation and OS	[20]
Src	Neck squamous cell carcinoma	122	-	Active expression correlates with advanced disease stage, lymph node metastasis and tumour recurrence	[21]
Src	Neuroblastoma	28	High expression (> 10%) in early tumours, low expression (< 10%) in aggressive tumours	High expression correlates with poor prognosis	[22]
ILK	HCC	57	Overexpressed in 36.9% (21/57); Overall expression levels were significantly higher (*P* = 0.004)	Elevated expression correlates with tumour progression	[23]
Paxillin	Ameloblastoma	44	Expression was mostly negative or very weak	The spearman correlation coefficient between FAK and paxillin expression was *r* = 0.43 (*P* = 0.002)	[15]
DLC-1	HCC	100	Significantly under-expressed (*P* < 0.0001)	Allelic losses ranging from 44 to 50%	[8]

Formalin-Fixed and Parrffin-Embedded tissue samples from patients with different tumours were analysed by immunohistochemistry to assess the expression levels of focal adhesion proteins and to explore the correlation between them and patients' clinical characteristics. *GIST* gastrointestinal stromal tumours, *OSCC* oral squamous cell carcinoma, *OS* overall survival, *HCC* hepatocellular carcinoma, *ILK* integrin-linked kinase

Recent studies have placed increasing emphasis on the interactions between the tumour microenvironment (TME) and tumour cells; the specific mechanisms of these interactions remain to be further explored. Focal adhesion, as an important adhesion structures for cell-ECM adhesion, play a key role in the interactions between tumour cells and the microenvironment. The aim of this review is to reveal the mechanism of focal adhesion as potential mediators of tumour metastasis. We first describe the molecular structure of focal adhesion and its intracellular assembly, and explore how tumour cells regulate its localisation, assembly and turnover. Next, we describe how focal adhesion mediates the behaviour of tumour cells and its role in promoting tumour metastasis. Finally, through a deeper understanding of these mechanisms, we envision potential therapeutic strategies against focal adhesion, especially how to intervene in tumourigenesis and progression by targeting focal adhesion-associated pathways, which opens up new possibilities for future tumour therapy.

### The overview of focal adhesion

Focal adhesion acts as a multimolecular complex that binds tumour cells to the ECM. This binding allows it to act not only as a physical anchoring structure, but also as a “mechanosensor" for the cell by transmitting mechanical signals (reviewed in [24, 25]). In recent years, there has been a growing body of research on focal adhesion in tumour cells. Studies have shown that many genes encoding focal adhesion proteins are mutated or overexpressed in many tumours. Meanwhile, the presence of a large number of tumour proteins in focal adhesion illustrates the importance of focal adhesion in tumour pathogenesis.

#### Structure of focal adhesion

**Fig. 1:**
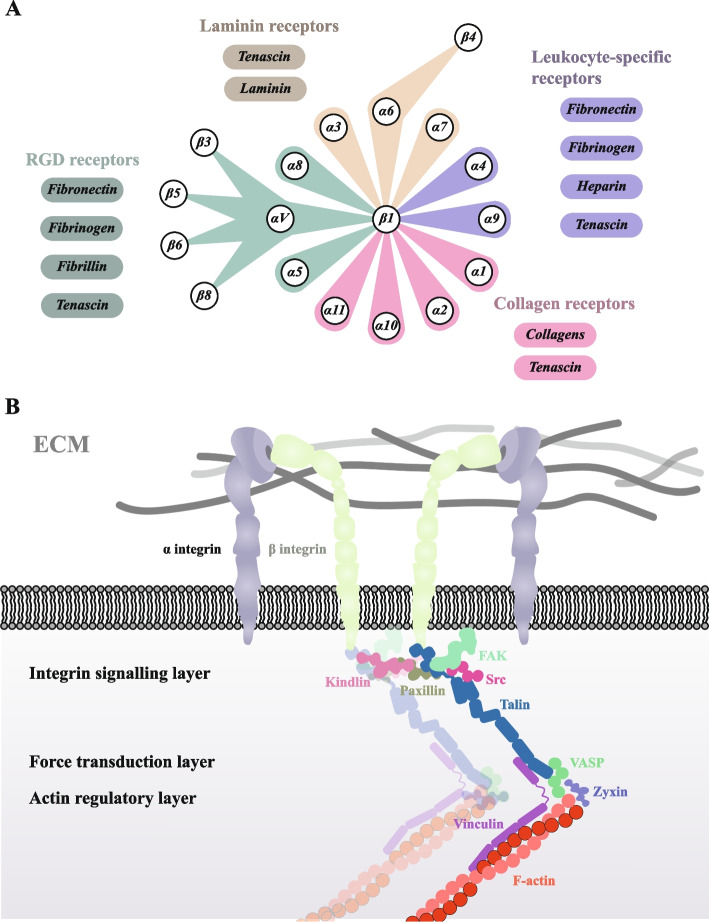
Connectivity of the extracellular matrix and intracellular structure of focal adhesion. **A** Focal adhesion-associated integrins can be categorised into four subgroups based on their distribution, ligand specificity, and function: RGD receptors, leukocyte-specific receptors, collagen receptors, and laminin receptors. These integrins are associated with specific sequences and regions of the extracellular matrix (shown as main matrix-integrin connections in this figure). **B** The intracellular molecular region of focal adhesion can be divided into three functional layers: integrin signalling layer (ISL), force transducer layer (FTL), and actin regulatory layer (ARL). Focal adhesion components tightly link the actin cytoskeleton, dominated by SFs, to the ECM through integrin heterodimers, forming a cell–matrix mechanistic whole

Focal adhesion is flat, wide structures with a thickness of approximately 50 nm, comprising many focal adhesion proteins [26]. These proteins represent the consensus adhesome with over 60 proteins as the core components, with the composition and abundance of the remaining proteins more finely regulated by the microenvironment [27]. Specifically, the protein exhibits dynamic changes in response to the demands of different cellular matrix environments (e.g. physical, chemical, and biological signals), as well as changes in cellular function (e.g. cytoskeletal organisation transformation). Despite its compositional instability, the primary mode of focal adhesion binding to the ECM relies on different types of integrin heterodimers, enabling the transmission of mechanical signals as a whole. In vertebrates, as Type I transmembrane glycoproteins, there are 24 different integrins, comprising 18 α-subunits and 8 β-subunits that interact non-covalently. Among them, the common focal adhesion-related integrins are α1β1, α2β1, α3β1, α4β1, α5β1, α6β1, α7β1, α8β1, α9β1, α10β1, α11β1, β4, αvβ1, αvβ3, αvβ5, αvβ6, and αvβ8 [28]. Different types of integrin heterodimers have been extensively described in various reviews as being linked to matrix components (Fig. 1A) [28–30].

Kanchanawong et al. resolved the low actin density core region in focal adhesion at approximately 40 nm and categorised it into three layers: the integrin signalling layer (ISL), force transducer layer (FTL), and actin regulatory layer (ARL) (Fig. 1B) [31]. Focal adhesion kinase (FAK) and paxillin in the ISL are responsible for collecting extra-matrix signals delivered by integrins α and β. Talin and vinculin in the FTL control the transmission of mechanical force, and the vasodilator-stimulated phosphoprotein (VASP)-zyxin complex in the ARL is involved in the connection between the focal adhesion and cytoskeleton. The cytoskeleton connected to the focal adhesion primarily comprises stressed fibres (SFs) formed by the assembly of F-actin [32]. These F-actin units are interconnected via α-actinin, fascin, and filamin [33–35], forming a solid stress structure in the focal adhesion. However, this layered model is not absolute, and proteins are not confined to a fixed hierarchy. For example, kindlin is located in different layers at different stages of focal adhesion assembly, thus regulating the dynamic assembly of focal adhesion [36, 37].

Recently, as our understanding of the molecular structure of focal adhesion has improved, new focal adhesion proteins have been discovered, and their role in tumours has received increasing attention. For example, ILK in the ISL forms the ternary ILK-PINCH-Parvin (IPP) complex by binding to integrin β1, PINCH, and parvin, and is reportedly significant in tumourigenesis, metastasis, and differentiation [38]. Caveolin-1 (Cav-1) is a focal adhesion protein co-localised with β-integrin and FAK [39, 40] and is crucial in signal transduction, tumour invasion, matrix remodelling, and matrix turnover [41–44]. However, there are many focal adhesion proteins, all of which cannot be discussed in this review; hence, we selected those most intensively studied for further exploration.

#### Focal adhesion protein-related gene expression and mutation

**Fig. 2:**
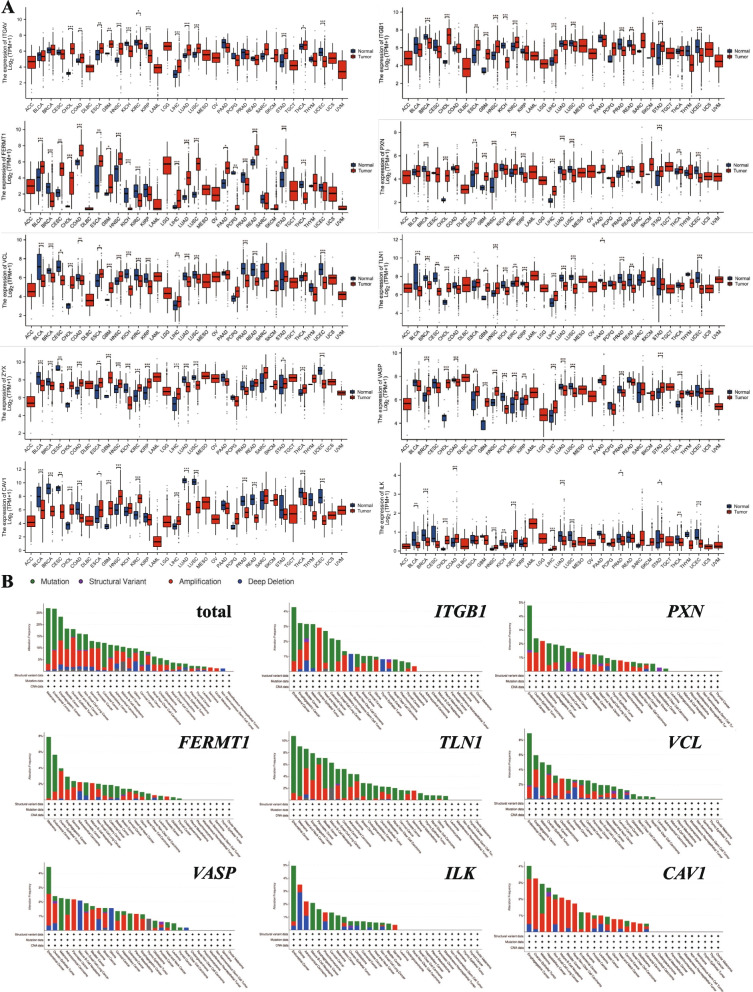
Focal adhesion proteins gene expression and mutation analysis. **A** Pan-cancer analysis of focal adhesion protein-related gene expression. Data were obtained from the TCGA database (https://portal.gdc.cancer.gov) containing RNA-seq data for 33 tumour items. These data were processed by STAR tool for comparison, extracted in TPM format and processed in log2(TPM + 1). Depending on the data characteristics, one-way analysis of variance (ANOVA) or Mann–Whitney U tests were applied and the *p*-value was corrected using the Benjamini–Hochberg method, with a *p*-value of less than 0.05 being considered a significant difference. Visual analyses were performed using R software and ggplot2, stats and car packages to demonstrate the distribution of focal adhesion protein-related gene expression in different cancer types. Software and R packages used: version R (4.2.1), ggplot2 [3.3.6], stats [4.2.1], car [3.1–0]. **B** Pan-cancer analysis of focal adhesion protein-related gene alterations. Using the cBioPortal platform (https://www.cbioportal.org), data from 32 cancer types in the TCGA pan-cancer study were selected for analysis. Alterations in eight focal adhesion protein-related genes, including *ITGB1* (integrin beta 1), *PXN* (paxillin), *FERMT1* (kindlin-1), *TLN1* (talin-1), *VCL* (vinculin), *VASP* (vasodilator-stimulated phosphoprotein), *ILK* (integrin-linked kinase), and *CAV1* (caveolin-1), were examined. Mutation types include point mutations (green), structural variants (purple), amplifications (red), and deep deletions (blue). These alterations were visualized and presented for the pan-cancer analysis

Aberrant expression of focal adhesion proteins is common in tumours. Numerous studies have reported that the overexpression of these proteins in various cancers correlates with poor tumour prognosis [45–49]. We downloaded and compiled RNAseq data from the The Cancer Genome Atlas (TCGA) database (https://portal.gdc.cancer.gov) for 33 tumour items and selected appropriate statistical methods for analysis. The results showed that most of the focal adhesion proteins were aberrantly expressed and predominantly overexpressed in a wide range of tumours (Fig. 2A). However, this pattern is not absolute, and some studies have shown that focal adhesion proteins are also under-expressed under certain circumstances [14, 22]. This suggests that the expression pattern of focal adhesion proteins may vary according to tumour type, stage or microenvironmental factors, reflecting the complexity of focal adhesion protein dysregulation in tumours, and also highlights the need for further studies to clarify the mechanisms and context of action of these variants.

Specifically, we processed the data through STAR alignment and provided the gene expression data in transcripts per million (TPM) formats. Meanwhile, in order to ensure data consistency and comparability among different samples, all data were processed by log2 transformation with the formula: log2(TPM + 1), which helps to reduce the bias of large differences in expression values and improve the stability of the data. Differential expression analysis focused on focal adhesion-related genes such as *ITGB1, PXN, FERMT1*, etc. Tumour types containing at least five samples were selected for the study. Statistical analyses were performed using stats and car from the R package, with one-way analysis of variance (ANOVA) for normally distributed data and the Mann–Whitney U test for non-normally distributed data. Multiple comparisons correction was done using the Benjamini–Hochberg method to control for false discovery rate (FDR). Gene overexpression was defined as a significant increase in gene expression in tumour samples compared to normal tissue, with the criterion that the TPM of tumour samples was at least twofold higher than that of normal tissue (i.e. log2(TPM + 1) ≥ 1) and a *p*-value of less than 0.05.

Latifeh Azizi et al. investigated the role of point mutations in talin in tumours by Catalog of Somatic Mutations in Cancer (COSMIC) database (https://cancer.sanger.ac.uk/cosmic) analysis and molecular dynamics [50]. Specifically, they screened 258 missense mutations through the COSMIC database and identified their positions in the talin structure. The pathogenicity of amino acid substitutions was predicted by the PON-P2 algorithm, structure/function effects were calculated using the BLOSUM62 and CBSM60 substitution matrices, and it was assessed whether the mutations caused a change in polarity in order to analyse the effect on the stability of the talin structure. Evolutionary conservation of amino acids was analysed in conjunction with ConSurf and it was determined whether the mutations were close to known ligand binding sites [50]. These mutations usually affect the assembly of focal adhesion and the migratory ability of tumour cells, which in turn has an impact on tumour progression. In addition, focal adhesion proteins such as integrin [51], FAK [52], Src [53], paxillin [54] and zyxin [55] have also been found to be present in tumours in a variety of mutated forms, and these mutations play an important role in tumourigenesis and progression. Specifically, the Y1389C mutation in talin affects the stability of its R7 and R8 structural domains, thereby enhancing the activity of the vinculin binding site [50]. In contrast, the *L2509P* mutation significantly affects the dimerization of talin, leading to a significant reduction in cell migration [50]. Meanwhile, through the analysis based on the cBioPortal database (https://www.cbioportal.org/), we selected eight key focal adhesion protein genes (*ITGB1*, *PXN*, *FERMT1*, *TLN1*, *VCL*, *VASP*, *ILK*, and *CAV1*) for mutation studies [56–58]. The results showed that the major types of mutations in focal adhesion proteins include point mutations (e.g., missense and nonsense mutations), structural variants (e.g., gene rearrangements, insertions, and deletions), amplifications (increase in copy number of the gene region), and deep deletions (significant reduction in copy number of the gene region). Analyses further revealed that mutations in the talin gene are more common in certain tumour types, particularly in Endometrial Cancer and Melanoma, suggesting that it may have an important role in the development and progression of these tumours. (Fig. 2B).

#### Bioinformatic analysis of focal adhesion proteins

In the RNAseq data analysis of focal adhesion proteins, RNA data were mainly obtained from TCGA database, NCBI Gene Expression Omnibus (GEO) archive (https://www.ncbi.nlm.nih.gov/geo/), cBioportal (http://www.cbioportal.org/), International Cancer Genome Consortium (ICGC) (https://dcc.icgc.org/) and other public databases. In addition, a number of databases corresponding to characterised cancer types exist. For example, for gliomas, there are also the Chinese Glioma Genome Atlas (CGGA) database (http://www.cgga.org.cn) and the Repository for Molecular Brain Neoplasia Data (REMBRANDT) (http://caintegrator-info.nci.nih.gov/REMBRANDT). These databases also provide relevant clinical data, thus offering the possibility to further explore the clinical relevance of focal adhesion proteins. The TCGA database also provides histopathological images including relevant H&E stains with corresponding pathological reports, clinical information and patient survival information. UCSC Xena database (https://xenabrowser.net/datapages/) provides phenotypic and survival data for a cohort of tumour patients. CCGA provides samples with different glioma grades, thus facilitating further prognostic studies.

After obtaining the raw data, it is usually necessary to go through data preprocessing steps to ensure the reliability and accuracy of the subsequent analyses. Firstly, quality control is an important part of preprocessing to safeguard the quality of data by excluding abnormal samples such as low count samples and abnormal mitochondrial gene ratios. In addition, tools such as RawVegetable [59], DoubletDecon [60], and Scrublet [61] are often used in order to identify and filter possible doublets. Samples can also be screened based on gene expression values, clinicopathological information, and survival data to optimise the accuracy of the analysed data. Next, normalisation of RNA-Seq data can effectively eliminate differences in sequencing depth and capture efficiency, making data comparable within and between samples [62]. Commonly used normalisation methods include CPM, TPM, RPKM, FPKM and DESeq2 size factor standardisation, followed by log(x + 1) transformation to further adjust the data distribution close to normal distribution [63]. Batch effects are a common problem in the analysis of large-scale RNA-Seq data, and therefore need to be corrected prior to downstream analyses using appropriate tools, such as RUVseq [64], SVASEQ [65], and tools developed for scRNA-seq data such as mutual nearest neighbours (MNNs) [66] and k- nearest-neighbour batch-effect test (kBET) [67]. Since RNA-Seq data are usually high-dimensional, dimensionality reduction of the data is required to facilitate analysis and visualisation. In diverse RNA-Seq studies, PCA is a classic and widely used dimensionality reduction method [63]. However, for scRNA-Seq data with complex structures, the effectiveness of PCA may be limited, for which more suitable methods such as T-distributed stochastic neighbour embedding (t-SNE) and uniform manifold approximation and projection (UMAP), which are more suitable for dimensionality reduction of single-cell data [47].By performing feature selection and dimensionality reduction operations, it can not only improve the computational efficiency of large-scale RNA-Seq data, but also help to focus on the key genes related to the research objectives, thus providing a solid foundation for subsequent in-depth analyses.

On the basis of the above data processing, combined with the joint analysis of focal adhesion protein expression and patient survival data, survival curves (e.g., Kaplan–Meier analysis) can also be plotted and the significance of differences can be calculated to further validate their prognostic relevance. For example, Rigiracciolo et al. collected FAK mRNA expression data from breast cancer patients from cBioportal, plotted Kaplan–Meier survival curves based on subgroups and calculated the significance of differences to validate their prognostic value [68]. The results showed that high FAK expression tended to predict a lower overall survival rate [68]. Multi-omics integration analysis (e.g., combined analysis of transcriptome with methylation group and proteome) can further deepen the understanding of the mechanism of focal adhesion proteins in tumours and their potential therapeutic targets, and provide new perspectives for personalized treatment of tumours. Meanwhile, the potential prognostic value of focal adhesion proteins in tumour patients and their functional mechanisms can be further determined using a series of statistical analyses. For example, Sun et al. used univariate Cox regression analysis and multivariate Cox proportional risk analysis to identify *PXN* expression as one of the prognostic indicators for patients [69]. Duong et al., on the other hand, established the correlation between the UNC13D expression values and the clinicopathological features of pancreatic cancer by ANOVA to further reveal the correlation between UNC13D and focal adhesion turnover coupling and pancreatic cancer cell migration, making it a newly identified prognostic factor in an independent cohort [70]. In addition, downstream analyses included a variety of bioinformatics approaches for a comprehensive exploration of the possible functions of focal adhesion proteins in tumours. Weighted gene coexpression network analysis (WGCNA) is widely used to construct gene co-expression networks to identify gene modules that are significantly associated with clinical features of tumour patients in order to explore the role of focal adhesion proteins in tumourigenesis, progression and metastasis [71]. Gene set enrichment analysis (GSEA) [72] and gene set variation analysis (GSVA) [73] can be used to detect the functional enrichment of focal adhesion protein-related genes and reveal their potential functions in signalling pathways, cell cycle regulation and metabolic processes. Functional enrichment analyses (e.g. GO and KEGG analyses) can be used to annotate the biological processes, cellular components, and molecular functions of focal adhesion protein-associated genes, thereby leading a more systematic understanding of their functional networks [74, 75]. Further analyses may also include exploration of the tumour immune microenvironment, such as assessing the relationship between focal adhesion protein expression and immune cell infiltration by tools such as CIBERSORT [76] and TIMER [77], in order to reveal its role in tumour immune regulation.

Mutations in focal adhesion proteins were screened and filtered by the COSMIC database, and GO and KEGG enrichment analyses were carried out in conjunction with the Functional Enrichment Tool [78, 79]. In addition, the distribution, function and clinical significance of focal adhesion proteins mutations can be comprehensively analysed by integrating COSMIC data with TCGA data, exploring the relationship between focal adhesion proteins mutations and patient survival or disease course through survival analysis (e.g., Kaplan–Meier analysis), or exploring these proteins mutation profiles and functional impacts through tools such as maftools and cbioportalR.

Analyses using a combination of bioinformatics tools have shown that multiple focal adhesion proteins serve as potential biomarkers at different stages of tumourigenesis and progression and are strongly associated with poor prognosis. For example, αvβ5 has been identified as a marker of metastasis in non-small cell lung cancer [80], whereas integrin α5 has been suggested to be an independent indicator of poor prognosis in oesophageal squamous cell carcinoma [81]. Preclinical and clinical studies have shown that integrin α2 can be used as a biomarker of treatment efficacy for E7820 [82]. Besides, the role of other focal adhesion proteins in a variety of tumours is gradually being investigated. In gliomas, paxillin overexpression is significantly associated with poor prognosis, mainly by promoting tumour cell proliferation and invasion [83]. The results of immune cell infiltration analysis also showed that paxillin overexpression was associated with a statistically significant infiltration of immune cells, such as B cells, making it an important biomarker of poor prognosis in ovarian [69]. In addition, talin [84, 85] and vinculin [86] have also been investigated as potential novel tumour biomarkers.

### Focal adhesion assembly in physiological conditions

**Fig. 3:**
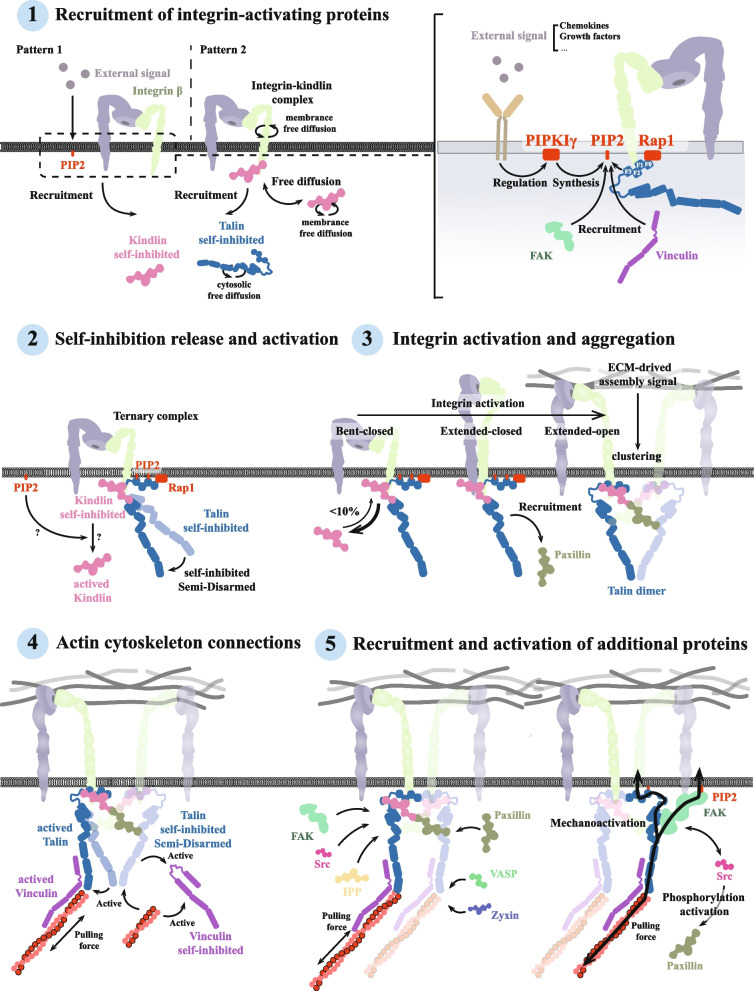
The intracellular assembly of focal adhesions. The intracellular assembly of focal adhesions can be broadly divided into five processes: (1) recruitment of integrin-activated proteins, (2) release of the autoinhibitory state and activation, (3) integrin activation and aggregation, (4) actin cytoskeletal attachment and (5) recruitment and activation of other focal adhesion proteins, and finally, assembly of the mature focal adhesions. Notably, these processes do not occur in strict chronological order and may occur in parallel. In addition, PIP2 plays an important role throughout the process, especially in step (1), where it promotes the recruitment and activation of integrin-activating proteins (e.g. talin and kindlin) under the regulation of PIPKIγ. In addition, PIP2 continues to regulate the recruitment of FAK in subsequent steps and interacts with Rap1 and other focal adhesion-associated proteins to further promote the assembly and maturation of focal adhesions

The assembly of focal adhesions is an orderly process that includes protein recruitment, conformational changes and activation. Individual proteins are assembled in a specific order, and perturbations at any stage may affect the overall assembly and function of focal adhesions. It is important to note that the sequence of these molecular events is not strictly linear, and may contain overlapping and synergistic effects of multiple stages. The overall process can be summarised in five steps: recruitment of integrin-activating proteins, release and activation of the autoinhibitory state, integrin activation and aggregation, actin cytoskeletal junctions, and recruitment and activation of additional focal adhesion proteins (Fig. 3).

#### Recruitment of integrin-activating proteins

Integrin activation requires the advance aggregation of a series of focal adhesion proteins at designated sites, which work together through synergistic action to promote integrin activation and aggregation. Current research suggests that this process can be accomplished with the synergistic action of talin and kindlin, but there are still other proteins, such as paxillin, are thought to play a role in this. This diverse recruitment mechanism allows focal adhesion to be assembled through multiple pathways under different conditions to adapt to the different needs of cells.

Although the N-terminal FERM structural domain of talin can activate integrins independently in the absence of ligand or mechanical force, this requires a high concentration of talin in the cytoplasm, which was not seen in actual cells [87]. It has been shown that activated members of the membrane-anchored Rap1 family of small GTPases can facilitate subsequent activation of integrins and focal adhesion formation by interacting with the F0 structural domain of talin [88]. In addition, the interaction of talin with membrane lipids, such as phosphatidylinositol 4,5-bisphosphate (PIP2), also plays an important role in talin recruitment [89–91]. Although Rap1 and PIP2 are independent regulators of talin recruitment to the membrane, recent studies have shown that the synergistic action of Rap1 and PIP2 significantly promotes talin recruitment and its subsequent activation [92]. Specifically, talin is recruited to the adhesion site through direct interaction with Rap1 via its F0 and F1 structural domains. Subsequently, talin further stabilises the localisation of its head domain at the adhesion site through interaction with PIP2, acting synergistically [92]. Notably, PIP2 is not uniformly distributed across the membrane, and its localised enrichment contributes to the directed assembly of focal adhesions, a process that may be dependent on the regulation of phosphotidylinosotol-4 phosphate 5 kinase γ (PIP5KIγ) [93]. PIP5Kγ is also localised in focal adhesion, and is able to direct focal adhesion assembly through its spatiotemporal and spatial-specific production of PIP2 [94]. Studies have also shown that talin recruits and activates PIP5Kγ, further promoting assembly and maturation of focal adhesions [95, 96]. In addition, chemokines and GFs regulate the targeted assembly of focal adhesions by modulating the local production and phosphorylation of PIP5Kγ [97–99]. However, according to the recent integrin-kindlin-talin ternary complex model, talin diffuses freely in the cytoplasm and is recruited only after kindlin forms a binary complex with the integrin, a process that does not depend on membrane [36]. This finding reveals the diversity of talin recruitment mechanisms and suggests that focal adhesion assembly can be regulated by multiple pathways under different conditions. The diverse recruitment mechanisms provide cells with a high degree of flexibility to rapidly regulate the assembly and dynamic remodelling of focal adhesions through multiple pathways in response to different external signals and internal demands, thereby effectively responding to complex physiological environments. This mechanism is important for the adaptive regulation of processes such as cell migration, signal transduction, and morphology shaping.

Unlike talin, kindlin is relatively understudied. Kindlin functions near the plasma membrane through free diffusion, rather than relying on a specific recruitment mechanism as talin does [36]. By binding to paxillin, it forms a “kindlin pool” in the integrin tail (mostly located in the ISL), increasing its probability of binding to the integrin and eventually being captured by focal adhesion [36]. Besides, its FERM structural domain contains the pleckstrin homology (PH) domain, which is able to bind to PIP2 and PIP3 on the membrane [100, 101]. This structural domain is essential for its recruitment from the cytoplasm to the vicinity of the membrane [101, 102].

#### Self-inhibition release and activation

When unassembled as focal adhesion, focal adhesion proteins, such as talin and kindlin, are normally in an autoinhibited state, ensuring that integrins are activated under specific conditions.

Talin is a multi-domain macromolecular protein that is mainly comprised of an N-terminal FERM domain and a C-terminal flexible rod-like domain [103]. In its N-terminal FERM structural domain, the interaction between F3 and R9 covers most of the focal adhesion protein-binding site, rendering talin autoinhibitory [104, 105]. Mechanical forces can disrupt this conformation, which in turn exposes multiple focal adhesion protein-binding sites and promotes focal adhesion growth and stabilisation. Meanwhile, recruited talin relies on its positively charged F2 (K268, K272, K274, R277 and K278) and F3 structural domains (K322 and K324) to interact with the cell membrane, favouring the binding of its F3 structural domain to the integrin NPxY motifs. However, its negatively charged C-terminal structural domain binds to the F2F3 structural domain via electrostatic interaction, thereby inhibiting its tight attachment to the membrane. The structural inhibition and charge inhibition described above constitute “dual inhibition” of talin. This dual inhibition mechanism ensures that talin is activated under certain conditions [106]. Overall, after talin is recruited through a PIP2-dependent mechanism, the negatively charged PIP2 binds to the F2F3 domain of talin electrostatically and pushes away the C-terminal domain by electrostatic repulsion, thus releasing the dual inhibition and activating talin [106].

Unlike talin, kindlin does not possess a rod-shaped structural domain, but it also includes atypical FERM structural domains containing the F1, F2, and F3 domains [107]. Kindlin-3 exists in an autoinhibited state via homo-oligomerisation [108]. In the trimeric state, the F2 subdomain of kindlin-3 is blocked by the PH domain of another kindlin-3 α2 helix, thereby blocking its binding to integrins [108]. At present, the mechanism of self-repression release of kindlin is not clear, but considering the role of the PH domain in this autoinhibitory state, it is possible that the autoinhibitory state of kindlin in cells might be regulated by effects on its PH domain [101]. However, this still requires further experimental validation to provide additional support.

It is difficult to clearly distinguish the sequence of events between the activation processes of talin and kindlin and that of integrins. This is because in the autoinhibited state, the integrin binding site of talin is closed, and only after it is released from this state can it bind to integrins and actin [109]. The force-induced unfolding property of talin suggests that it requires an anchoring site (usually located in the integrin tail), to unfold the autoinhibitory state in response to external forces [103]. The multiple structural domains of talin have different reactivity to mechanical forces, and each structural domains will sequentially unfold under increasing mechanical loads [110]. Thus, when talin in the self-inhibited state is recruited to the vicinity of integrins, it is first deregulated from initial self-inhibition by a non-actinin-dependent mechanism in order to bind to the integrin tail. This initial binding of talin could contribute to the further release of actin-dependent autoinhibition, culminating in full activation of talin. This release process does not occur transiently, but rather progressively.

#### Integrin activation and aggregation

Integrins exist in three conformational states at the cell membrane, bent-closed, extended-closed, and extended-open [37, 111]. This conformational change regulates the affinity of integrins for the ECM, allowing them to receive assembly signals from the ECM [111–113]. Meanwhile, changes in the intracellular structural domains of integrins further modulate their affinity for ligands, facilitating the recruitment and assembly of subsequent focal adhesion proteins [114]. Focal adhesion proteins, such as PIP2, kindlin, and talin, are synergistically involved in integrin activation, through interactions with membrane-neighbouring components, and together promote focal adhesion assembly [101, 115–117].

With the advancement of single-molecule positioning and tracking technology and protein observation techniques, such as NMR, it is now believed that the integrin–kindlin–talin triad forms a transient dynamic complex in an assembly–disassembly cycle [36, 118]. This model sufficiently explains the complex synergistic role of kindlin and talin in integrin activation [36]. Specifically, integrins diffuse freely in the plasma membrane in a bent conformation. The binary binding mode between integrin and kindlin occurs with a higher success rate compared to that when integrin, talin, and kindlin meet simultaneously and bind correctly. Freely diffusing kindlin binds to integrins, forming a binary complex that changes the conformation of the β-subunit, further facilitating talin binding and activating the integrins. This is consistent with earlier studies [119]. However, talin binding can weaken the ability of kindlin to bind to integrins, resulting in a greater tendency for kindlin to dissociate from the ternary complex, but this is not an absolute situation. This tendency to dissociate allows only a small fraction (< 10%) of integrins to form ternary complexes stable enough to withstand high mechanical forces [118]. Under appropriate conditions, these small amounts of activated integrins gradually assemble into the mature focal adhesion complex.

Integrin aggregation is an important mechanism for extending the lifespan of activated integrins, which in turn become focal adhesion precursors (reviewed in [117]). Preliminary research suggests that kindlin is thought to play a role in this process [120]. Follow-up studies further revealed that the density of integrin clusters formed varies with different matrix stiffnesses, as follows: on softer matrices, integrin clusters are spaced at ~ 200 nm, whereas on stiffer matrices, integrin clusters are denser and more spaced at ~ 60 nm [117, 121]. It is noteworthy that a sufficient density of integrin clusters is a necessary prerequisite for talin dimer formation [122]. Talin dimer formation is dependent on two neighbouring activated integrins. Specifically, with the formation of the integrin–talin–kindlin ternary complex, paxillin is also recruited to the integrin upon partial activation of talin. It facilitates the formation of talin dimers by linking to the kindlin of the neighbouring integrin to form the talin–paxillin–kindlin bond, which promotes the aggregation of the two integrins that form talin dimers. ultimately promoting the full activation of integrins and the assembly of focal adhesions [123].

Thus, talin and kindlin promote integrin aggregation while altering integrin conformation for activation. This process relies on a high matrix stiffness to ensure sufficient force loading to unify the integrin "outside-in" and "inside-out" activation models.

#### Actin cytoskeleton connections

Talin has three actin-binding sites (ABS1, ABS2, and ABS3). Of these, ABS2 is located in the R4-R8 structural domain, whereas ABS3 is located at the C-terminus [124, 125]. These three actin-binding sites play different roles in different stages of the assembly of focal adhesions: nascent adhesion depends on the binding of ABS3 to the actin cytoskeleton [126, 127], whereas this maturation is usually accompanied by the exposure of ABS2 and its binding to the actin cytoskeleton [128]. ABS2 is more deeply embedded in the autoinhibitory conformation of talin than ABS3; after factors such as PIP2 release the initial autoinhibitory state, ABS3 is first exposed to actin binding. As the load applied to talin increases, the autoinhibitory state is further lifted and ABS2 is exposed, allowing for tighter binding to actin and driving the maturation of focal adhesions.

During under load, additional focal adhesion protein-binding sites of talin are exposed, among which vinculin binding sites (VBSs) located near ABS2 are also revealed [129, 130]. In vitro studies have shown that, after VBS exposure, vinculin binding locks the conformation of talin, contributing to the continued exposure of ABS2 and further stabilisation of the structure of focal adhesion [128]. This binding also has a buffering effect on talin forces [110].

#### Recruitment and activation of additional focal adhesion proteins

The assembly of focal adhesions involves the recruitment and activation of a variety of focal adhesion proteins, and the current study reveals the early mechanism of action of some of the key proteins (e.g. talin and kindlin). However, it is not clear whether the recruitment sequence of other focal adhesion proteins has a similar temporal hierarchy, and further studies are necessary.

Focal adhesion proteins are usually classified as having force-dependent and phosphorylation-dependent recruitment mechanisms. This classification emphasises the coordination of mechanistic signals with biochemical signals during the assembly of focal adhesions. For example, one of the recruitments of FAK depends on the biological force generated by myosin II contraction [131]. This contractile force allows integrins to bind to fibronectin (FN) synergy site, thereby enhancing integrin–ECM associations [132]. Besides, epidermal growth factor (EGF) signalling also recruits FAK to the focal adhesion through phosphorylation of the integrin β-subunit Y1526 [133]. This dual recruitment mechanism, coordinating mechanical and biochemical signalling has been experimentally demonstrated for major focal adhesion proteins such as vinculin [110, 128–130, 134–136], paxillin [137–139], zyxin [137, 138, 140], and Src [141, 142]. Among these forces, there are diverse sources, including intracellular forces such as myosin II contraction [131, 143–145], myosin VI contraction [146], retrograde actin flow [147, 148], and extracellular forces with an ECM–integrin origin [149–151]. Moreover, in this process, the recruitment of membrane PIP2 [152] and focal adhesion protein-dependent recruitment through interactions [153] must not be neglected. Activation of focal adhesion proteins is often regulated by phosphorylation. Zaidel-Bar et al. proposed a "switch model" to explain the effects of paxillin phosphorylation and dephosphorylation on the assembly of focal adhesions [154]. Meanwhile, the tensile activation properties of some focal adhesion proteins make focal adhesion proteins mechanosensor (pN level) for the detection of integrin tension. When a tension of 56 pN was applied to the integrin, the size of the focal adhesion formed was found to be approximately twice as large as that at 12 pN. This process is accompanied by an increase in the level and activity of FAK-pY397 phosphorylation [155]. These two activation pathways are not independent of each other and often function together to completely activate FA proteins.

Membrane composition PIP2 also has a unique role in this process. It anchors FAK to the plasma membrane by binding to FAK FERM structural domain and facilitates the release of FAK autoinhibition, activating FAK autophosphorylation site Y397[152]. The FAT structural domain of FAK also enhances its plasma membrane localisation and tension transduction by binding to paxillin. Under the actin cytoskeleton pull of ~ 25 pN applied, the conformation of FAK changes further, exposing its active site and Src phosphorylation site, completing the full activation of FAK [156]. This cascading activation process reflects the multilevel signalling regulation mechanism in the assembly of focal adhesions and helps to fine-tune the regulation of FAK activity.

Taken together with the current study on focal adhesion protein recruitment and activation, except for talin and kindlin, other focal adhesion proteins might exhibit the same temporal hierarchy during this process, and the exact chronological order is difficult to distinguish. However, in order to deepen the understanding of the assembly of focal adhesions, an attempt can be made to roughly sort out its chronological order based on experimental evidence.

Considering the scaffolding role of paxillin, one could argue that it is paxillin that acts earlier in this temporal hierarchy, but this view is not fully established. Paxillin can be recruited in a kindlin/talin-dependent or non-kindlin/talin-dependent manner and undergoes initial phosphorylation in response to stimulation via the mechanical force exerted by ECM–integrins. This mechanical force-mediated stimulation leads to low levels of paxillin phosphorylation and high levels of dephosphorylation, with phosphorylation predominating early. When equilibrium is reached, the overall paxillin phosphorylation-to-dephosphorylation ratio in the adhesion zone determines the course of further phosphorylation and the recruitment of FAK, which binds to it after a certain threshold has been reached. FAK is perhaps recruited to the plasma membrane in response to different factors, including integrins and PIP2, and is initially released from its autoinhibitory state and its autophosphorylation. In summary, two tension-conducting structures appear at this point in the plasma membrane, integrin–talin (vinculin)–actin filaments and PIP2–FAK–paxillin–actin filaments, with the former acting as the primary force-conducting structure and the latter playing a role in the full activation of FAK. After full activation of FAK, multiple focal adhesion proteins are phosphorylated by forming a FAK/Src kinase complex with Src. Upon actin cytoskeleton attachment, zyxin and VASP act as actin regulators that are recruited to focal adhesion in response to actin cytoskeleton-transduced forces.

#### Some outstanding issues

Although protein observation techniques such as plasmonic scattering microscopy (PSM) [157] and single-fluorescent-molecule imaging tracking (SMT) techniques [158, 159] have made it possible to observe the movement of specific proteins in the focal adhesion with the interactions, but the current update of knowledge on major focal adhesion proteins is still insufficient. Focal adhesion, as a protein complex, has been relatively understudied due to its diverse protein components leading to the complexity of interactions. Although studies have been conducted to progress from single focal adhesion proteins to specific series of regulatory processes, the lack of holistic studies may lead to cognitive bias. Therefore, further studies on the dynamic assembly view of focal adhesion focal adhesion remain a crucial issue for the future.

Focal adhesion serves as an anchor between the cell and the ECM, and its importance in mechanosensing is increasing. Pathological processes are often accompanied by dysregulation of mechanical signalling, and how focal adhesion responds to this dysregulated mechanical signalling or participates in causing an imbalance in the mechanical environment, the mechanical properties of focal adhesion proteins are of increasing interest. Current studies have shown that many focal adhesion proteins are mechanically responsive, for example, the mechanical responsiveness of zyxin affects its cytoplasmic-nuclear localisation [160]. This response bypasses the mechanotransduction of the actin cytoskeleton, suggesting that the role of force in the cell is not only "physical". Talin, the core of mechanotransduction in focal adhesion proteins, exhibits different lengths under different force loads to avoid extreme force fluctuations within the cell, thus maintaining intracellular homeostasis [110]. All these phenomena reflect the importance of mechanotransduction in focal adhesion assembly, and further detailed characterisation is required in the future.

Studies have also shown that there is a complex crosstalk mechanism between integrins and GFs and that focal adhesion appears to be a meeting point for biochemical and mechanical signals (reviewed in [161]). Biochemical signals in the environment have been studied to regulate the assembly of focal adhesions by phosphorylating focal adhesion proteins [133], but there is still a lack of understanding of how mechanistic signals interact with them. Cells are usually affected by both signals in vivo, and thus an in-depth exploration of the coordinating mechanisms between the two in the assembly of focal adhesions remains an important research direction.

Focal adhesion under different conditions presents different morphologies, showing a dynamic view of assembly-disassembly. This reflects the dynamic nature of focal adhesion turnover, i.e., focal adhesion is not only a responder to the environment, but also an adaptor to the environment. Focal adhesion can better perform its function by regulating the dynamic turnover to meet the demands of different environments. This is particularly evident during cell motility [162]. Therefore, further studies on the dynamic turnover of focal adhesion and its role in other biological processes are also worth exploring in depth.

### The regulation of focal adhesion in tumour cells

In tumour cells, focal adhesion in pathological states is often abnormally numerous and structurally disordered, leading to dysregulated signalling [163–166]. These abnormalities increase the migration and invasiveness of tumour cells, which is further exacerbated by hyperphosphorylation or aberrant expression of specific focal adhesion proteins. Not only that, under the regulation of the malignant tumour stroma, multiple cell types (e.g., immune cells, endothelial cells, stromal cells) may also show similar deregulation of focal adhesion, which may collectively drive tumour progression (reviewed in [25, 167, 168]).

#### Regulation of focal adhesion by mechanical force

In tumour cells, both external stresses originating from the ECM and internal mechanical forces generated by the actin cytoskeleton tend to be higher than in normal cells [169, 170], this higher force aids in the assembly and turnover of focal adhesions.

"Catch-bond" and "slip-bond" are two different types of protein–protein interactions, the main difference being the change in bond strength under the influence of mechanical forces. A catch-bond is an interaction that strengthens with increasing force, in contrast to the slip-bond found in most protein–protein interactions, where the bond weakens as the force increases [171]. Tumour cell integrins resist high solid stress from the tumour ECM by forming a catch-bond with the ECM. Catch-bond formation is dependent on a mechanical force-driven conformational change, that shifts the integrin from a low-affinity state to a high-affinity state, thereby enhancing integrin binding to the ECM [171]. The integrin maintains an open and stable conformation at loads of ~ 3 pN and breaks at loads exceeding 100 pN [172–174]. The integrin-ECM interaction time was significantly prolonged in the loads range of 10–30 pN, which is consistent with catch-bond properties [174, 175]. This extended lifespan facilitates the assembly of focal adhesions, as the temporarily immobilised integrins provide time and space for recruitment and activation of focal adhesion proteins [159]. In addition, the synergistic site of FN, although it does not directly affect the binding of integrins to FN, regulates the phosphorylation of FAK-Y397 through enhanced adhesion, which in turn facilitates the assembly of focal adhesions [176]. This enhanced adhesion is a resistance force, non-tractive force that contributes to the efficient conduction of mechanical forces [172].

The formation of catch-bonds by integrins not only enhances the binding of tumour cells to the ECM, but also facilitates the assembly of focal adhesions and the perception and transmission of ECM signals. Meanwhile, the conversion of catch-bond to slip-bond facilitates the weakening of cell adhesion, which leads to tumour metastasis. In tumour cells, the oncogene *DLC1* tends to be inactivated, a process that not only upregulates the expression of FAK and talin, but also inhibits RhoA signalling, which in turn attenuates the contractility of the actin cytoskeleton [177]. This inhibition makes the catch-bond more susceptible to shift to a slip-bond, leading to focal adhesion breakage, which in turn promotes tumour cell migration. Besides, when the contractile force of the actin cytoskeleton is transmitted to the focal adhesion, it often results in micrometre-scale backward sliding of the focal adhesion relative to the ECM, known as “frictional slip” [178]. This friction contributes to the maturation of focal adhesions and enhanced integrin-ECM interactions [178].

In tumour cells, internal loads lead to conformational changes in some focal adhesion proteins and produces distinct effects under enhanced intracellular loads. Each domain of the talin's rod structure has an independent mechanical response, and a load of ~ 25 pN is required to fully deploy the talin (when a force of ~ 4 pN/s is applied) [110]. This explains why integrin activation requires a force of only about 3 pN, whereas the force conducted on it often exceeds 100 pN. The tensile state of talin in isolation cannot be maintained and rapidly returns to its original state when subjected to forces less than 3 pN [110]. However, in stabilized focal adhesions, due to the combination of vinculin and talin, the tensile state of talin can be maintained to a certain degree of ductility even under smaller loads (~ 2.5 pN). This mechanical stability may be related to the fact that vinculin enhances the mechanical toughness of talin and the exposure of the ductile domains, thus facilitating its function in focal adhesion. Correspondingly, in stable focal adhesions, vinculin undergoes a force of ~ 2.5 pN, but this load may be higher in the early stages of assembly, which may be related to force-dependent conformational activation of other focal adhesion proteins [179]. In addition, retrograde flow of actin acts on focal adhesion proteins through repeated stretching cycles, inducing conformational changes that in turn promote assembly and disassembly of focal adhesions (reviewed in [180]).

Focal adhesion showed significant differences in cell formation on rigid versus elastic substrates, which was closely related to the resistance generated by the cytoskeleton and the tension on focal adhesion [181, 182]. Cells tend to form more SFs and a greater number of larger, more stable focal adhesion on rigid matrices compared to elastic ones [151, 183–186]. This phenomenon is primarily driven by the contractility of cells. Focal adhesion transmits not only intracellular contractile forces, but also solid stresses of the matrix, and thus play an important role in balancing these oppositely directed forces. The optimal stiffness for the formation and maturation of focal adhesions in tumour cells depends on the mechanical balance between intracellular contractility and matrix stiffness [182]. The optimal matrix stiffness fort the formation of focal adhesions and migration varies between different tumour cells, which is closely related to the differences in contractility of different tumour cells and is particularly significant in different microenvironments [187–189].

#### Regulation of focal adhesion by signalling molecules

In addition to focal adhesion proteins phosphorylation due to the FAK/Src kinase complex during the assembly of focal adhesions, other intracellular signalling pathways regulate its formation, localisation, size and stability by phosphorylating focal adhesion proteins. In particular, the MAPK signalling pathway, protein kinase C (PKC), Abelson kinase and PI3K/AKT signalling pathways are particularly important in this process [190–198]. These signalling pathways influence the dynamic properties of focal adhesion and the biological behaviour of cells by interacting and regulating the phosphorylation status of focal adhesion proteins, which in turn facilitate cellular adaptation to the microenvironment.

In recent years, the role of mechanosensitive signalling pathways in tumour biology has attracted much attention, such as the Hippo signalling pathway [199], Integrin signalling pathway [200], Wnt signalling pathway [201, 202] and TGF-β signalling pathway [203]. These signalling pathways help tumour cells to adapt to the mechanical features imposed by ECM deterioration by converting cellular mechanical signals into biochemical signals with the formation of adapted focal adhesions. Specific adaptations are manifested in the enhancement or diminution of focal adhesions, depending on the location and functional requirements of focal adhesions. For example, the matrix in the anterior part of the tumour cells is usually denser, and the front of the tumour cell needs to be subjected to stronger solid stresses [204]. During tumour cell migration, the anterior end of the cell needs to form a stronger focal adhesion to anchor cell protrusions and propel cell motility [169]. This mechanism reveals how tumour cells use mechanical signals to regulate their behaviour to adapt to the demands of the dynamic microenvironment.

Yes-associated protein (YAP), a major effector of the Hippo signalling pathway, induces tumour cell mechanosensing regarding cell volume and shape by interacting with downstream TEA structural domain family members (TEAD) [199, 205]. As an important oncogene, YAP plays a key role in several aspects of tumour progression, especially in the highly rigid tumour stromal environment, where YAP often exhibits high activity and overexpression [206, 207]. High levels of YAP affect focal adhesion kinetics by activating downstream transcription factors that regulate gene expression in tumour cells. It has been shown that the YAP-TEAD complex promotes the phosphorylation of FAK-Tyr397 through the transcription of *THBS1*, thereby driving the formation of focal adhesions and the invasiveness of breast cancer cells [163]. Furthermore, ChIP-Seq analysis of YAP revealed that YAP directly regulates the expression of focal adhesion proteins-related genes, which contributes to the assembly of focal adhesions [208]. Mechanical loads imposed by the tumour ECM affect the stability of the actin cytoskeleton and focal adhesion by altering the shape and volume balance of tumour cells. Overexpression of YAP further enhances actin cytoskeleton stabilisation by activating Rho GTPase signalling and maintains the stability of focal adhesion in breast cancer cells [208].

Multiple integrins are highly expressed in tumours and are closely associated with tumour metastasis [209]. Unlike other mechanosensitive signalling pathways, the activation of the integrin signalling pathway occurs in the focal adhesion, and its downstream is similar to the intracellular regulatory mechanisms of the focal adhesion. Integrins aggregate upon binding to ECM ligands and phosphorylate FAK via non-ligand tyrosine kinases, thereby activating the phosphatase activity of the FAK/Src kinase complex [210]. The FAK/Src kinase complex activates the phosphatase activity of the FAK/Src kinase complex by phosphorylating a variety of intracellular focal adhesion proteins in tumour cells including paxillin [139, 191, 211, 212], p130cas [211, 213, 214], vinculin [211], etc. At the same time, the FAK/Src kinase complex also affects cytoskeletal dynamics in tumour cells by phosphorylating Rho, Rac and Cdc42, a regulation that contributes to the formation of focal adhesions [210]. In addition to interactions with RTK, activation of the integrin signalling pathway in tumour cells also modulates the downstream effects of ILK (reviewed in [215]). This is largely dependent on the formation of the IPP complex by ILK, which regulates actin cytoskeleton dynamics and is involved in the regulation of focal adhesions [216]. For example, IPP enhances the stability of focal adhesion in a non-catalytically active manner by promoting F-actin bundling [217].

#### Ion regulation of focal adhesion

Among the many ions, calcium ion influx is particularly important for the ionic regulation of focal adhesions in tumour cells. Mechanosensitive ion channels fulfil their key function in the assembly and dynamics of focal adhesions, mainly by regulating calcium ion inward flow [218]. Elevated calcium ion levels and distribution polarity can influence its formation, stabilisation and disassembly by modulating the actin cytoskeleton or focal adhesion components within tumour cells, thereby impacting focal adhesion dynamics.

Calcium ions affect the hydrolysis of focal adhesion proteins by activating calpain. For example, calpain is able to cleave key focal adhesion components such as FAK [219], talin [220–222] and paxillin [223], affecting its turnover process. The fragment formed by cleavage of the N-terminus of talin by calpain can bind to Smurf1 (an E3 ubiquitin ligase) and subsequently be ubiquitinated for degradation, thus promoting the disassembly of focal adhesions [222]. Similarly, paxillin produces a ~ 55 kDa fragment similar to δ-paxillin in the presence of calpain. The fragment acts as an endogenous inhibitor that inhibits the maturation of focal adhesions and hinders tumour cell migration [223]. Overall, calpain-mediated focal adhesion protein hydrolysis both promotes assembly and disassembly of focal adhesions, forming a bi-directional regulatory mechanism, a balanced mechanism that enables tumour cells to adjust their migratory and invasive properties in a changing microenvironment.

Increased calcium ion concentration in tumour cells significantly affects the dynamic properties of focal adhesion by regulating the activity of several protein kinases, which have an impact on the phosphorylation of focal adhesion proteins. The appearance of a single intracellular calcium spike can influence the focal adhesion turnover by regulating FAK phosphorylation [224]. This is further supported by the fact that the mechanosensitive calcium channel piezo1 is stably localised to mature focal adhesion and regulates its growth and turnover [225]. Pyk2 is activated in response to stimulation by intracellular calcium ion concentrations, thereby regulating the dynamics of focal adhesions in breast cancer cells [226, 227].

Tumour cells usually maintain sensitivity to mechanical signals by forming a gradient in the distribution of calcium ions, i.e. lower levels of calcium ions at the front of migration and higher levels of calcium ions at the back, to support the polarity of the direction of migration [228]. Tumour cells increase focal adhesion strength at the pseudopodium by small amplitude and small diameter calcium waves, which do not lead to dramatic fluctuations in local calcium concentration, helping to maintain the spatial polarity of calcium ions and fine-tune the dynamic properties of focal adhesion [229]. Fine regulation of calcium ions in time and space thus induces differences in actin cytoskeleton dynamics, allowing tumour cells to develop different focal adhesion dynamic properties before and after migration (reviewed in [230]).

#### Regulation of focal adhesion by cell membrane composition

Increased lipid rafts in tumour cells strongly correlate with the degree of malignancy [231, 232]. Lipid rafts are specialised lipid bilayer structures enriched with specific lipids (e.g. cholesterol and sphingolipids) that play an important role in the regulation of membrane dynamics [233]. This property allows some integrins (e.g., α4β1) to aggregate in them, facilitating the assembly of focal adhesion [234]. In tumour cell migration, lipid rafts regulate integrin β1 and β3 subunit aggregation and drive the assembly of focal adhesion in the cell leading edge [235, 236]. Meanwhile, lipid rafts provide a more suitable membrane environment for adhesion proteins assembly, which may be due to the more ordered lipid structure [237, 238] or it contributes to the formation of integrin homo-oligomers [239]. This is due to the fact that lipid rafts have a thicker membrane thickness (10–200 nm) compared to non-lipid raft regions, which helps to stabilise the activated conformation of integrins and thus promotes integrin activity [240].

During metabolic reprogramming of tumour cells, the composition of lipid rafts changes dynamically, further enhancing their malignant characteristics. Acyl-CoA synthetase long-chain family member 4 promotes the metastasis of breast cancer cells by remodelling the lipid composition of lipid rafts, affecting the compartmentalisation and activity of integrins and promoting their aggregation [241]. In non-small cell lung cancer, cholesterol depletion displaces Src from the membrane to the cytoplasm, resulting in focal adhesion disassembly and subsequent inhibition of tumour cell migration [242]. This suggests that the spatial localisation of focal adhesion proteins in lipid rafts is critical for adhesion maturation.

Caveolae are another important lipid structure, and reduction is often associated with poor tumour prognosis [243, 244]. It is similar in composition to lipid rafts, but predominantly present as vesicular structures of 50–100 nm, where cav-1 play a key role in its formation and stabilisation [245]. The high degree of membrane order in the focal adhesion region is mainly contributed by the ordered membrane components induced by phosphorylated cav-1 [238, 246]. Ordered membranes contribute to stable binding of focal adhesion proteins [246]. As a junction protein, cav-1 promotes integrin signalling by interacting with focal adhesion proteins, regulating the assembly and turnover of focal adhesions by stabilising FAK and Src. Phosphorylated cav-1 can promote the turnover of focal adhesions in breast cancer cells by stabilising FAK [246]. It also can activate Rho GTPases via Src, which in turn remodels the actin cytoskeleton, thereby affecting the formation of focal adhesions and the migratory capacity of tumour cells [247, 248]. Unphosphorylated cav-1 is usually located at the posterior end of migrating cells, whereas phosphorylation of its Tyr14 contributes to its relocation to the anterior end of migrating cells and participates in assembly [249]. Thus, dynamic regulation of cav-1 phosphorylation is critical for cell polarity and the turnover of focal adhesions. Caveolae is also involved in turnover by regulating integrin aggregation and endocytosis. In synergy with lipid rafts, it promotes aggregation and activation of integrin β-subunits, thereby facilitating cellular assembly of focal adhesions on rigid matrix [250]. However, phosphorylated cav-1 regulates the turnover by mediating integrin endocytosis, contributing to the dissociation of cell–matrix adhesion [39, 251].

Plasma membrane microstructural domains, along with PIP2, also play a significant role in focal adhesion regulation. PIP2, as a key signal transduction molecule, is closely related to tumourigenesis and development [252, 253]. In adhesion regions, PIP2 was highly enriched, which is mainly attributed to the local concentration of PIP5KIγ at focal adhesion [95, 96]. The relationship between PIP2 and lipid rafts is still under investigation, and there is evidence that lipid rafts may promote their transmembrane accumulation through PIP2 recruitment [254], and the ordered lipid structure within lipid rafts seems to be more favourable for PIP2 production and metabolism [255]. Enrichment of PIP2 promotes the recruitment of focal adhesion proteins within ordered membrane compartments and further enhances focal adhesion assembly by facilitating focal adhesion protein oligomerization. (e.g. vinculin [256–258] and FAK [152]).

### Focal adhesion creates conditions for tumour cell migration and invasion

**Fig. 4:**
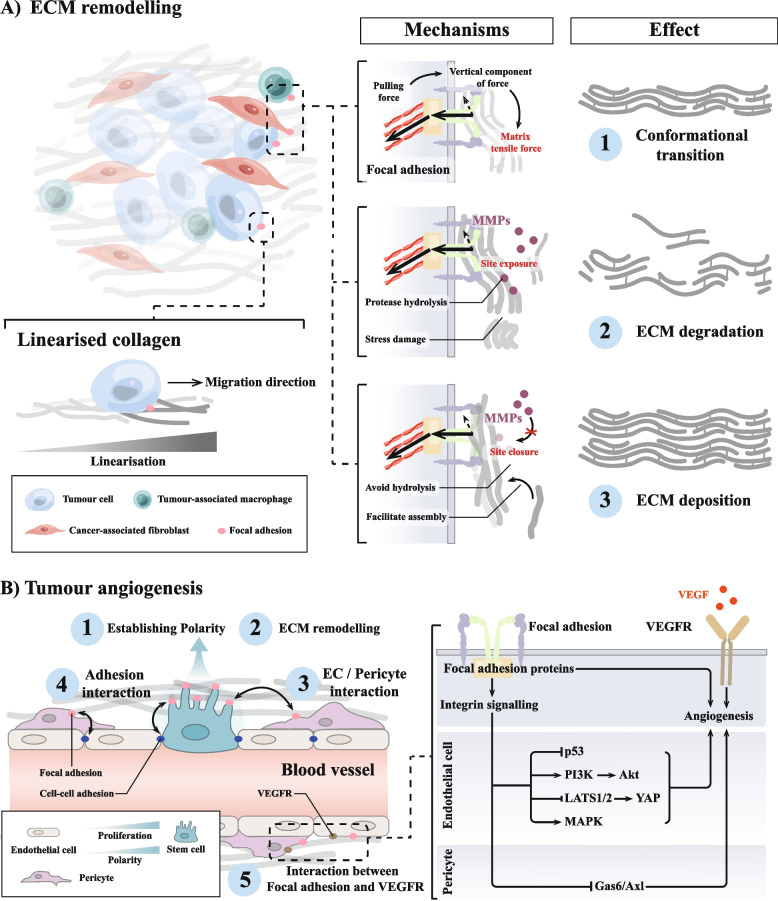
Focal adhesion creates conditions for tumour cell migration and invasion. **A** Focal adhesion can directly remodel the matrix by exerting biological forces on the ECM through integrin-mediated mechanical actions. The process of tumour matrix remodelling via focal adhesion mainly involves conformational changes in extracellular matrix (ECM) components, matrix degradation and matrix deposition, forming a complex tumour matrix environment. At the same time, the degree of collagen linearisation in the tumour matrix intensifies towards tumour cell migration, which may be related to the biological forces of focal adhesion action. **B** Focal adhesion is involved in tumour angiogenesis. Focal adhesion in endothelial cells (ECs) plays important roles in tumour angiogenesis, including (1) establishment of endothelial cell polarity, (2) vascular ECM remodelling, (3) ECs/pericyte interaction, (4) adhesion interactions, and (5) interactions between focal adhesion and vascular endothelial growth factor (VEGF) signalling. Moreover, the intracellular signalling pathways activated by focal adhesion in different cell types (especially ECs and pericytes) showed different effects, revealing the complexity of focal adhesion in regulating tumour angiogenesis

Aggressive tumours develop through a complex series of steps; among them, the migration and invasion of tumour cells are considered to be central components of the invasion-metastasis cascade [259]. In vivo, the ECM surrounding solid tumours is usually hard [260]. Large amounts of cross-linked and deposited collagen, and other ECM proteins form fibroproliferative ECM structures [260, 261]. These linear collagens provide a “highway” for tumour cells to migrate through the parenchyma to the vascular system. This process is often accompanied by phenomena such as tumour angiogenesis and dysregulation of the cytokine environment, which create conditions for tumour cell migration and invasion. Tumour cell migration is also accompanied by a large number of changes to the ECM, which focal adhesion, as a cell–matrix adhesions, not only sense, but even participate in (Fig. 4).

#### Focal adhesion-mediated ECM remodelling

The ECM is a complex collection of matrix proteins, enzymes, and glycoproteins that provides a suitable “hotbed” for cell growth [262]. It is tightly regulated under normal conditions and is capable of regulating a wide range of normal cellular behaviours [263]. When ECM homeostasis is altered, it is usually manifested as a change in matrix composition and matrix properties; this process is called matrix remodelling. Abnormal matrix remodelling may trigger a range of pathological responses, including tumours [264, 265]. Tumour-associated matrix remodelling paves the way for tumour cell migration into the tumour vasculature system, and is crucial for tumour cell migration and invasion.

Biomechanics play a crucial role in matrix remodelling, especially in the TME with imbalanced mechanical signalling. Cell-ECM interactions are key to tumour matrix remodelling, as discussed by Jelle et al. [266]. Biological forces delivered by integrins act on ECM components to alter the conformation of ECM proteins, affecting them deposition and degradation, which termed “force-mediated ECM remodelling” [265]. The "molecular clutch" theory explains how the flow of the actin cytoskeleton is translated into traction forces on the ECM via focal adhesion. This theory is mainly used to study the mechanism of cell migration to explain the generation of specific friction between cells and their microenvironment. When the actin cytoskeleton is attached to the ECM via focal adhesion, actin monomers polymerise at the barbed end of pre-existing actin filaments, pushing the cell membrane forward to form protrusions. At the same time, the thrust generated by this polymerisation reacts back on the ECM through the focal adhesion, resulting in traction [267]. Various devices have been developed to measure this mechanical force, such as FRET biosensors [268], molecular tension fluorescence microscopy [269] and reversible shearing DNA probe [270], among others.

Mechanical loads can metamorphose the ECM, leading to matrix deposition and hydrolysis. Specifically, mechanical forces applied to collagen fibres impede the conformational transition of collagenous protofibrils and prevent their hydrolysis [271]. Trimeric collagen under mechanical force stretching is more susceptible to binding to matrix metalloproteinases (MMP), mediating further cleavage [271, 272]. Besides, the disruption of the basement membrane (BM) is also an important process in tumour ECM remodelling. Tumour cells and other cells, such as cancer-associated fibroblasts (CAFs), are able to undergo force-dependent BM remodelling through mechanical forces generated by their contraction [265, 273, 274]. Such matrix remodelling would facilitate the shaping of migration-friendly matrix features, thus promoting tumour cell migration. Roca-Cusachs et al. described a variety of mechanical parameters between the ECM and integrins, outlining the manifestation of ECM metamorphosis in response to integrin-mediated force transfer (reviewed in [180]). There is no direct experimental evidence on whether focal adhesion is capable of producing these effects, and more experiments are based on integrin studies. However, it is undeniable that focal adhesion, as the most representative integrin-mediated cell–matrix adhesion structure, also plays a key role in force-mediated matrix remodelling.

Helvert et al. found by atomic force microscopy nanoindentation (AFM) that during cell migration, the mechanical forces of actin contraction and integrin delivery are sufficient to cause compaction and densification of collagen fibres at the leading edge of the cell, thereby inducing matrix rearrangement, a process that is dependent on integrin-mediated cell–matrix adhesion [275]. In breast cancer cell migration experiments, deformation of collagen fibres around focal adhesion was observed by second harmonic generation (SHG) imaging, suggesting that focal adhesion-transmitted biomechanics can induce changes in the local ECM microstructure to adapt to the migratory demands of tumour cells [276]. Further in vivo imaging studies also validated this idea [276]. The linearised collagen network enhances the formation of cell protrusions through mechanotransduction, thereby increasing the efficiency of tumour cell motility [277]. Stress–strain analysis of the collagen network showed that the mechanical behaviour of the collagen network gradually transitioned from a linear response to an exponential change in response to the contractile force exerted by the contracting cells. This phenomenon reveals that the Poisson effect of collagen fibres under the mechanical action imparted by focal adhesion enables the cell contraction force to significantly increase fibre densification at lower mesh densities [278–280]. This mechanical adaptation process plays a key role in the stiffening and densification of the matrix, thus further contributing to tumour progression.

**Fig. 5:**
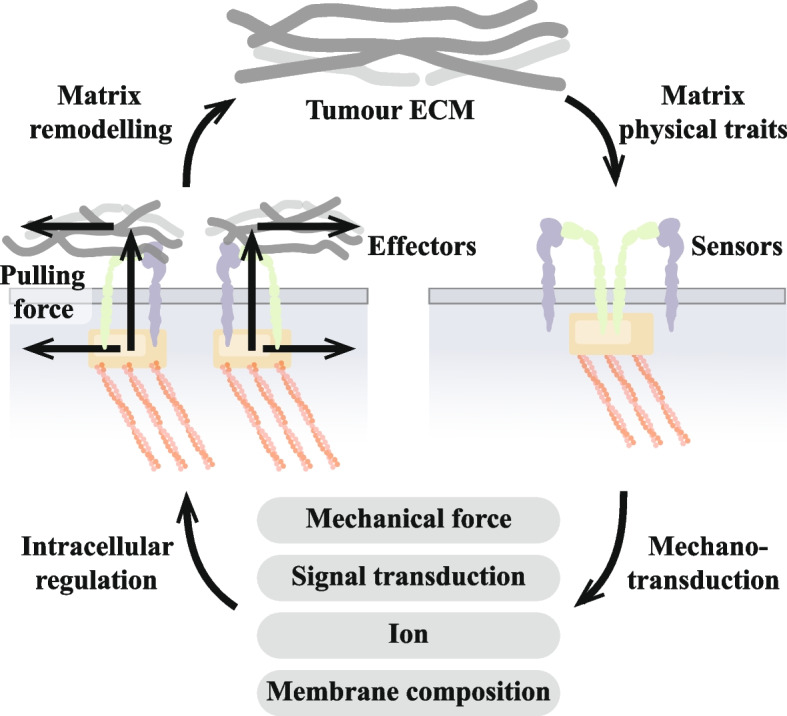
Focal adhesion-mediated disruption of positive feedback in matrix remodelling. In this process, focal adhesion not only acts as a receptor, sensing the matrix information of the deteriorating tumour extracellular matrix (ECM) and regulating its own assembly, but also acts as an effector, participating in the remodelling of the tumour ECM, further exacerbating its deterioration. This positive feedback loop promotes the formation of the final tumour ECM

The physical microenvironment of a tumour includes a range of biomechanical features, such as physical microstructural, matrix stiffness, and solid stress [281]. Matrix remodelling develops new mechanical features in tumour development that influence cellular behaviour through mechanotransduction pathways, which in turn have a further impact on the physical properties of the matrix. In this process, focal adhesion acts both as an effector of tumour matrix remodelling and as a receptor for sensing mechanical signals of matrix deterioration (Fig. 5). In a breast cancer study, Calvo et al. found that matrix stiffness activates YAP in CAFs, which regulates MYL9/MLC2 to contract the actin cytoskeleton and transmit forces via focal adhesion to enhance matrix stiffness [282]. This finding suggests that a positive feedback loop in the TME, where the deteriorating matrix influences cellular behaviour through focal adhesion mechanotransduction, further involving force-mediated remodelling. However, this hypothesis requires further validation by more in vivo and ex vivo experiments.

#### Focal adhesion-mediated tumour angiogenesis

In contrast to physiological angiogenesis, tumour blood vessels are often considered tortuous, leaky, and disorganised [283]. The angiogenic process is driven by the need to supply oxygen and nutrients through neovascularisation to sustain growth as the tumour increases in size to 1–2 mm [284]. Although multiple mechanisms drive tumour angiogenesis, vascular sprouting is still regarded as the main tumour angiogenesis pathway [285, 286]. Currently, several in vivo and ex vivo models have demonstrated that focal adhesion deficiency can leads to impaired vasculature neogenesis, termed “focal adhesion-deficient vasculature” [287–291]. In this process, focal adhesion regulates endothelial cells (ECs) polarity, proliferation and interactions with pericytes, influencing the formation of the tumour vascular system.

Tip cells, specialised ECs generated by competition among EC subtypes during angiogenesis, direct vascular sprouting [292]. Focal adhesion influences the morphology and direction of elongation of tip cells, guiding sprouting of tortuous tumour vessels. It aggregates between the adherent and non-adherent zones, and the structure of contractile SFs across non-adhesive strips enhances the lateral stability of the cell, limiting the ability to elongate perpendicular to the pattern direction, thereby driving ECs to elongate along the pattern direction [293]. Meanwhile, focal adhesion activates the integrin signalling pathway by sensing matrix signals from tumour vascular neogenesis, further regulates the direction of tip cells elongation. Integrin deficiency leads to the downregulation of the polarity protein Par3, resulting in the loss of cell polarity required for neovascularisation [294]. Loss of polarity may further affect the regulation of VE-calmodulin and other intercellular adhesion molecules, interfering with Slit and Robo, and affecting lumen formation [295]. In addition, tip cells acquire environmental cues for neovascularisation through their filopodia to enable directed migration [296]. Neovascular adhesion in the filopodia requires the recruitment of multiple focal adhesion proteins to form stable focal adhesions for cell migration. Itraconazole exerts anti-neovascular effects by altering the localisation of IP6K1 and enhancing the interaction of IP6K1 with 5-InsP7, which inhibits the turnover of focal adhesions and cell migration capacity [297].

Compared to normal vessels, tumour vessels are poorly sealed, which is closely related to the interactions between focal adhesions and intercellular adhesions in ECs. Focal adhesion-deficient vessels exhibit increased permeability, due to the role of focal adhesion in linearising intercellular junctions [298, 299]. This mechanism may be associated with increased actin polymerisation and enhanced actin network stability [288]. It is generally accepted that cell–cell adhesion is usually considered to be regulated by cell–matrix adhesion; while the integrity of cell–cell adhesion in ECs is crucial for preventing tumour vascular leakage [300, 301]. Existing studies have mainly focused on the direct role of focal adhesion in regulating ECs. The crosstalk regulatory mechanism between focal adhesions and cell–cell adhesions in tumour angiogenesis remains to be further explored by future experiments.

Immediately beneath the tip cells is a group of proliferative ECs, termed stem cells, whose division promotes the growth of vascular sprouts, as well as the formation of tumour vasculature. Deficiency or overexpression of the talin affects the activation of integrins, which in turn affects the proliferative capacity of ECs via the MAPK/ERK signalling pathway [299]. Besides, its overexpression maintains the tension-transducing structure of integrin-talin-actin filaments and exhibits a relatively low vascular defect profile [299].

Interaction between ECs and pericytes is crucial for tumour angiogenesis, where disruption in this interaction severely compromises vascular stability and permeability, reflecting typical tumour vascular characteristics [302]. In tumour vascular system, pericytes usually highly express NG2, a surface molecule essential for EC-pericyte interaction through its co-localisation with integrin β1 [303]. Deficiency of NG2 leads to loss of collagen VI anchoring, which, in turn, triggers tumour vascular matrix remodelling [304]. Additionally, NG2 is able to influence pericytes recruitment through the cis- or trans-activation of integrins, leading to the dysregulation of interactions between ECs and pericytes [305–307]. Integrin/FAK signalling at focal adhesion in pericyte-mediated tumour angiogenesis is poorly investigated. Negative regulation of FAK activation in tumour angiogenesis has been demonstrated, which may be dependent on Gas6/Axl signalling [308]. In addition, phosphorylation of the FAK Y861 site affects tumour cell apoptosis and vascular remodelling by regulating paracrine factors, such as tumour necrosis factor (TNF)-α and SDF1 [309].

#### Focal adhesion-mediated dysregulation of the cytokine environment

Cytokines are low molecular proteins (6–70 kDa) secreted by a variety of cells that act by regulating the communication network of cells [310]. Based on their functions, cytokines can be classified as interferons, transforming growth factors (TGFs), TNFs, and interleukins [310]. In the TME, the secretion and action of cytokines are often out of control, resulting in tumour cells being in a state of unbalanced cellular communication (reviewed in [311–314]). These uncontrolled cytokines create an inflammatory and immunosuppressive TME, which provides favourable conditions for tumour cell migration and invasion. Here, we describe how focal adhesion leads to this phenomenon, using the example of dysregulation of the GFs environment.

The ECM serves as a reservoir for cytokines. Cytokines stored in the ECM can only exert their biological functions once they are released into the surrounding microenvironment in a free form. Mechanical deformation of the ECM is thought to facilitate cytokine release. For example, TGF-β is usually present in the ECM in a potential form bound to latency-associated peptide (LAP) [315]. When the actin cytoskeleton of tumour cells contracts, the contractile forces can mediate LAP deformation via integrins, which, in turn, causes the release of TGF-β [28, 203, 316, 317]. In the TME, TGF-β levels tend to be elevated and are involved in malignant events such as tumour matrix fibrosis; therefore, TGF-β could be considered central to cytokine signalling imbalances [318, 319]. Although there is no direct experimental evidence for the role of focal adhesion in this process, it is possible that the biologic force of the actin cytoskeleton on the ECM via integrins relies on focal adhesion as a molecular clutch. Further, integrins involved in this process, such as αvβ6 and αvβ8, have been suggested to be integrin heterodimers on focal adhesion [28]. However, force-dependent activation of TGF-β appears to be largely dependent on specific types of integrin heterodimers (e.g., αvβ6 and αvβ8), whereas a number of low-affinity interacting integrin heterodimers (e.g., αvβ1, αvβ3, and αvβ5) have been found to play a relatively weak role in this process [320]. Due to the diversity of integrin species in focal adhesion and the dynamic changes in their expression and distribution in response to cellular functional demands, there are challenges in validating the effectiveness of specific focal adhesion in force-mediated TGF-β activation.

Besides, focal adhesion provides an enrichment site for focal adhesion proteins, which promotes their activation in a mechanosensitive manner. Activated focal adhesion proteins regulate the behavioural pattern of tumour cells by interacting with the corresponding receptors and responding to extracellular cytokine signals in an unconventional manner. This pattern of enrichment and activation combines mechanotransduction and chemo-transduction in tumour cells, making focal adhesion the intersection of the two. Platelet-derived growth factor (PDGF) receptor activity can only be maintained at high levels in a mechanically stressed matrix; integrin-ECM mechanical linkages contribute to the formation of such a hard matrix [321]. EGFR enhances the activity of Src kinase within the local focal adhesion by binding to FAK, thereby promoting actin contraction and inhibiting adhesion spot kinetic processes [322]. In addition, integrins, as receptors for fibroblast growth factor (FGF-1) and insulin-like growth factor (IGF-1), are involved in the regulation of downstream signalling pathways and play an important role in the proliferation and metastasis of tumour cells [323–325].

Although existing studies have revealed the critical role of various focal adhesion proteins (e.g. integrins, FAK) in the formation of an imbalanced tumour cytokine microenvironment (reviewed in [161, 326, 327]), experimental data directly demonstrating that focal adhesion as a whole regulates cytokine release are still relatively limited. The available evidence mainly relies on indirect observations of the role of focal adhesion-related signalling molecules, while the functional mechanism of focal adhesion as a holistic structure in the regulation of cytokine release has not been sufficiently investigated. In addition, the role of focal adhesion in the dysregulation of GF signalling in abnormal tumour cells has been relatively well investigated [328–330], but its relevance to the function of focal adhesion as a holistic structure still needs to be further explored.

To further reveal the role of focal adhesion in TGF-β release, a series of precise experimental designs can be explored. Firstly, fluorescent labelling of key focal adhesion proteins (e.g. talin, vinculin) and storage forms of TGF-β (e.g. potential forms bound to LAP) was combined with live cell microscopy techniques to observe the spatiotemporal co-localisation of focal adhesion dynamics with TGF-β release during cell migration or contraction. Secondly, AFM was used to apply controlled mechanical loads on tumour cells or ECM to monitor the dynamic changes of focal adhesion proteins in real time. Meanwhile, focal adhesion protein tension was quantified using mechanical sensors (e.g., FRET probes) to assess the relationship between focal adhesion protein stress and FRET signals. In addition, ELISA and immunofluorescence methods were used to quantify TGF-β release levels in cell supernatants and explore the correlation between the dynamics of focal adhesions and TGF-β secretion. To further validate the role of focal adhesion proteins in this process, the expression or function of focal adhesion proteins can be inhibited by targeted knockdown using CRISPR/Cas9 technology or by using specific protein inhibitors, and the changes in the release levels of GFs (e.g., TGF-β) in the simulated tumour ECM environment before and after the interventions can be compared by ELISA, immunofluorescence, or Western blot techniques. With these experimental designs, researchers can systematically elucidate the potential mechanisms by which focal adhesion regulates TGF-β signalling in the tumour microenvironment.

### The role of focal adhesion in tumour metastasis

**Fig. 6:**
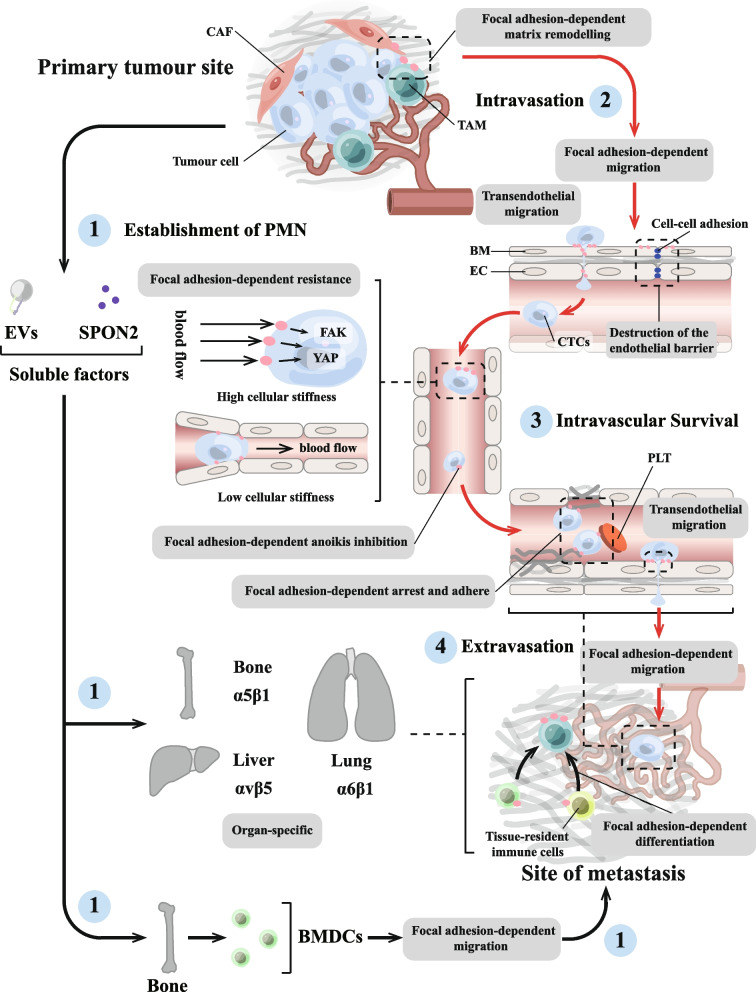
Focal adhesion in tumour metastasis. Tumour metastasis involves five steps: (1) establishment of the pre-metastatic niche (PMN), (2) intravasation, (3) intravascular survival, and (4) extravasation. In process (1), immune cells migrate to secondary growth sites via focal adhesion, with extracellular vesicles (EVs) containing focal adhesion components determining organ specificity. focal adhesion-dependent differentiation generates tumour-associated immune cells that promote metastasis through matrix remodelling, angiogenesis, and the creation of an immunosuppressive microenvironment. In process (2), tumour cells migrate from parenchymal tissues to the vascular system via focal adhesion, exerting force on the basement membrane (BM) to disrupt it and facilitate entry. Dysregulation of focal adhesion dynamics in endothelial cells (ECs) further disrupts the vascular barrier, aiding tumour cell invasion. In process (3), circulating tumour cells (CTCs) adapt to different environments by modulating focal adhesion properties and mimic focal adhesion signals to avoid anoikis. In process (4), CTCs identify the extravasation site through focal adhesion-mediated recognition of multiple ECMs and migrate from the vasculature to the metastasis site in a focal adhesion-dependent manner. In the figure, the boxes on the grey background represent the cellular behaviours involved in focal adhesion

Tumour metastasis is a multistep process that can usually be divided into three overlapping phases: dissemination, dormancy, and colonization [331]. During these phases, tumour cells play an important role in various key aspects through focal adhesion sensing and pulling multiple ECM, such as fibrotic ECM and endothelial ECM. Bioinformatics analyses have shown that focal adhesion plays a crucial role in driving multiple aspects of the metastatic process in multiple types of tumours [332–336] (Fig. 6).

#### Establishment of a pre-metastatic niche

The pre-metastatic niche (PMN) usually forms prior to tumour metastasis, providing the “soil” for tumour cells to colonise [337, 338]. The establishment of the PMN is dependent on three main factors: primary tumour-derived constituents, tumour-driven recruitment of bone marrow-derived cells (BMDCs) and adaptive matrix remodelling [339]. Focal adhesion plays an important role in this process, participating in cell adhesion and migration signalling and enhancing the perception and regulation of the matrix environment by a variety of cells, including immune cells.

Organ-specific metastases are to some extent dependent on soluble factors secreted by the primary tumour cells. For example, exosomes expressing integrin α5 have been shown to induce bone metastases in breast cancer [340], whereas exosomes expressing integrin αvβ5 are more often associated with liver metastases [341]. The mechanism of this organ-specific metastasis may involve integrin-containing extracellular vesicles (EVs) by promoting focal adhesion assembly and interaction with the ECM [341]. In addition, differences in ECM composition and properties in different organs may further contribute to the organ-specificity of metastasis (reviewed in [342]). For example, knockdown of *SERPINB1*, a brain niche-specific ECM proteins, in breast cancer cells significantly reduced brain metastasis [343]. This finding is consistent with the functional characterisation of focal adhesion, as cells bind to specific ECM components via different integrin heterodimers for niche-specific adaptation. In conclusion, the specificity of organ metastasis may arise from the synergistic interaction of integrin heterodimers in focal adhesion with organ-specific ECM proteins, thus unifying integrin-mediated assembly and niche-specific ECM binding within the same mechanistic framework. In addition, the ECM components contained in EVs, such as transglutaminases, FN and laminin (LN), provide potential support mechanisms for the dynamic assembly of focal adhesions [344, 345]. Specifically, FN-integrin complexes and other adhesive fragments in EVs play a key role in the assembly of focal adhesions, a dynamic adhesion structure, and the presence of these inclusions enhances the assembly efficiency [346]. Thus, the information carried by EVs related to the assembly of focal adhesions may determine the site of PMN formation, which further contributes to organ-specific metastasis of tumours.

PMN can be broadly divided into four main stages during its dynamic evolution: early PMN, mid PMN, late PMN and metastatic niche [347]. Tissue-resident immune cells and matrix cells are actively involved in the establishment of early and intermediate PMN through focal adhesion-matrix interactions in response to soluble factors secreted by tumour cells [347]. Tissue-resident immune cells are usually organ-specific, a property that is thought to play a key role in the organotropism of tumour metastasis, but more direct evidence is still needed to confirm this. For example, tissue-resident macrophages include hepatic Kupffer cells [348], bone osteoblasts [349], lung macrophages [350], and brain microglia [351], among others. It has been shown that EVs carrying integrin αvβ5 are able to selectively bind Kupffer cells, which further determines the specificity of liver metastasis [341]. Besides, integrin-binding sialoprotein (IBSP), a phosphorylated and glycosylated matrix protein containing an integrin-binding RGD (Arg-Gly-Asp) sequence, is strongly associated with poor prognosis and bone metastasis in a variety of tumours, including lung, breast and prostate cancers [352–354], and focal adhesion may play an important role in this process [353, 355, 356]. Recent studies have shown that IBSP further promotes bone metastasis in lung adenocarcinoma by promoting the differentiation of bone tissue-resident osteoclasts, leading to early osteolysis and creating favourable conditions for remodelling the bone microenvironment [357].

In late PMN, a variety of immune cells are progressively recruited and migrate into the local microenvironment [338]. Similar to tumour cells, immune cell migration is also dependent on focal adhesions to sense matrix signals and provide mechanical traction [358–362]. SPON2 secreted by tumour cells promotes transendothelial migration of plasma-derived monocytes by activating the Pyk2 signalling pathway [363]. This process is accompanied by both cytoskeletal remodelling of macrophages and the dynamic assembly of focal adhesions, and blocking the function of integrin β1 significantly reduces M2-tumour-associated macrophage (TAM) infiltration [363]. In addition, macrophages activate the SFK-FAK/CSF-1R signalling pathway through focal adhesion with FN, which enhances their migration [364]. At the same time, recruited BMDCs also rely on focal adhesion to establish interactions with FN, further exerting immunosuppressive functions [347]. This dynamic process not only attracts more immune cells to be recruited and accumulated, but also accelerates the final maturation and evolution of PMN.

Tissue-resident and recruited immune cells promote adaptive matrix remodelling and increased vascular permeability through the interactions of focal adhesions with surrounding tissue cells and the ECM. For example, during the course of liver fibrosis, Kupffer cells induce activation and matrix remodelling of hepatic stellate cells (HSCs) in response to soluble factors secreted by tumour cells [365]. In turn, focal adhesion activation of HSCs favours the expression of relevant ECM proteins (e.g. FN) [366]. Pre-metastatic matrix remodelling is the starting point for PMN establishment and creates favourable conditions for subsequent processes. During liver fibrosis, overexpression of FN promotes the recruitment of BMDCs and the formation of an immunosuppressive environment [365]. This matrix remodelling also provides conditions for the colonisation of circulating tumour cells (CTCs). Specifically, FN can promote invasion and migration of lung cancer A549 cells by activating FAK signalling and modulating MEK1/2 and PI3K signalling pathways, thereby enhancing MMP9 expression [367]. Vascular permeability is also increased in this process, which includes both protease-dependent and focal adhesion-dependent events [368, 369]. How focal adhesion is involved in the formation of vascular leakage has been reviewed in detail previously. Overall, these tumour-associated immune cells shape the channels for tumour cell invasion by secreting protein hydrolases or through force-dependent mechanisms, which facilitates the subsequent extravasation of CTCs.

Tissue-resident and recruited immune cells gradually differentiate into tumour-associated phenotypic cells in the TME and play a key role in promoting inflammatory responses and immunosuppression in PMN. In studies of lung metastasis from HCC, increased expression and morphological changes of FN and focal adhesions were closely associated with the shift of fibroblasts to a tumour-associated phenotype [370]. This shift not only promotes the formation of inflammatory PMN in the lungs, but also triggers organ-specific metastatic processes [370]. In particular, the differentiation capacity of TAM is highly dependent on the interaction between focal adhesions and FN. Focal adhesion-α5β1 integrin activates the p72Syk signalling pathway, enhancing macrophage migration and differentiation [371]. In addition, SPON2 secreted by tumour cells further exacerbates the immunosuppressive state by activating the Pyk2 signalling pathway and promoting macrophage differentiation towards M2-TAMs [318]. In a study of triple-negative breast cancer, inhibition of the BRD4/c-Myc and focal adhesion signalling pathways by synergistic use of JQ1 and VS-6063 effectively reduced the infiltration of Ly6G⁺ MDSCs and significantly inhibited tumour growth and spread [372]. These studies suggest that the role of focal adhesion in the phenotypic differentiation and functional regulation of immune cells is crucial, and the aberrant activation of its signalling pathway may be an important mechanism to promote tumour progression. Meanwhile, these mechanisms also provide new ideas for focal adhesion as a potential therapeutic target.

#### Intravasation

Endocytosis is the process by which tumour cells enter the vascular system from the tissue in situ. Matrix remodelling and tumour neovascularisation are key steps in this process, and the role of focal adhesion has been described in detail previously. The fibrotic and rigid ECM provides a “highway” for tumour cells to migrate from parenchymal tissues into the vascular system, significantly enhancing their migration and endocytosis [264, 373]. This “highway” is manifested in multiple ways, including increased turnover of focal adhesions [374, 375], enhanced actin cytoskeletal dynamics [376], and less resistant ECM barriers [377, 378].

**Fig. 7:**
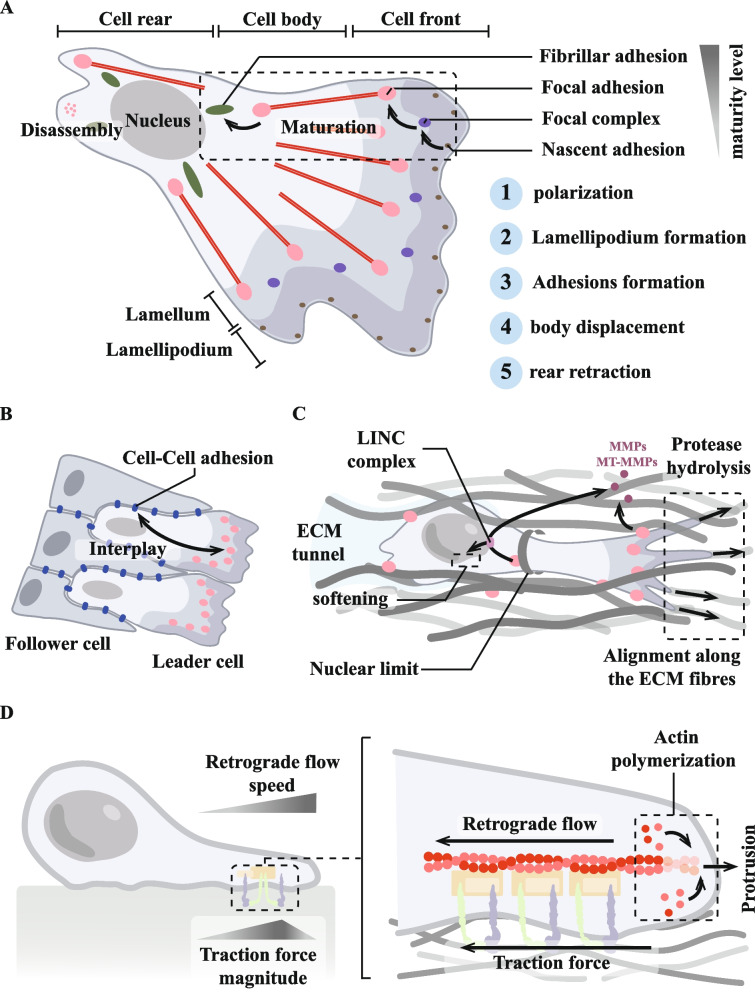
Focal adhesion in tumour cell migration. Mesenchymal migration is divided into five processes: (1) polarisation, (2) lamellipodium formation, (3) adhesion formation, (4) body displacement, and (5) rear retraction. In cells undergoing mesenchymal migration, the distribution of focal adhesions shows asymmetry and exhibits a certain regularity: it matures from the anterior part of the cell to the posterior part, where it is finally disassembled. **B** During collective migration, focal adhesion synergistically promotes the collective migration of cell clusters through interactions with cell–cell adhesion. **C** The tumour microenvironment in vivo is usually very complex and focal adhesion determines the direction of cell migration by aligning collagen fibres. As the bulky nuclei of tumour cells restrict their movement in the extracellular matrix (ECM), focal adhesion regulates membrane type-1 matrix metalloproteinase (MT1-MMP) activity through mechanosensing while softening the nucleus to promote cell body translocation. In addition, focal adhesion-regulated MT1-MMP secretion was able to form tunnels in the ECM, further promoting subsequent cell migration. **D** Focal adhesion acts as a “molecular clutch” that facilitates the maturation of focal adhesion by generating retrograde actin flow through the assembly of the actin cytoskeleton at the cell front. During this process, focal adhesion also generates a traction force on the ECM, which pushes the cell body forward

Focal adhesion plays an important role in single-cell migration and collective migration of tumour cells (Fig. 7). Single-cell migration refers to the ability of a cell to move through a range of behaviours, such as polarisation, lamellipodium formation, adhesion formation, body displacement, and rear retraction [379]. This process is known as mesenchymal migration and usually requires mature intracellular SFs, as well as strong cell–matrix adhesion [380]. As an important representative of cell–matrix adhesion, focal adhesions anchor the tumour cell to the matrix, thus enabling the tumour cells to sense the matrix and gain motility, which is central to cell movement [381, 382]. It has been shown that the leading edge of migrating tumour cells typically assembles nascent adhesion sites which subsequently mature into focal adhesions [383]. These mature adhesions undergo disassembly at the posterior part of the cell, and this dynamic assembly-disassembly is closely associated with morphological changes in cell migration [384]. At the same time, the leading edge of the migratory direction is usually accompanied by a massive turnover of adhesions, resulting from the continuous reorganisation of the actin cytoskeleton in response to migratory signals [162].

In collective migration, cell clusters typically consist of leading and following cells [385, 386]. Leading and following cells bind through cell–cell adhesion, and coordinate the overall migration of the cell cluster. It is currently believed that the basic mechanisms of single-cell migration also apply to collective migration, but leading and following cells require additional steps in their respective migration processes. This leads to more complex cell–cell and cell–matrix connections. For example, coordinating the coherent polarity and movement consistency of cell clusters requires complex intercellular connections. Cell–cell adhesion interacts with focal adhesion to enable tumour cells to integrate information from neighbouring cells and matrix for coordinated collective migration.

Fibrotic ECM promotes endocytosis of tumour cells by modulating the dynamics of focal adhesion, inducing tumour cells to assume a basal-like phenotype [387]. Tumour cells with a basal-like phenotype have a greater contractile capacity and are able to disrupt the basement membrane through focal adhesion-mediated mechanical actions, resulting in more efficient access to the vascular system [387, 388]. In addition, imperfect barrier function of the tumour vasculature contributes to transendothelial migration of tumour cells, and studies have shown that tumour endocytosis is often accompanied by a temporary increase in local vascular permeability [389]. As discussed previously, vascular sealing is closely linked to the regulation of endothelial cell focal adhesion in terms of tight junction linearisation. Dysregulation of focal adhesion dynamics in ECs not only weakens the integrity of the vascular barrier, but also creates favourable conditions for tumour cells to cross the vessel wall.

In summary, focal adhesion is involved in the process of transendothelial migration of tumour cells at multiple levels not only by participating in tumour cell motility, but also by modulating tumour cell phenotype, disrupting the BM, and affecting the vascular barrier function of ECs, which ultimately contributes to the formation of CTCs.

#### Intravascular survival

When tumour cells are dislodged from the primary tumour site or metastatic site and enter the peripheral circulatory system, they are termed CTCs. CTCs face multiple challenges in the bloodstream that contribute to their low survival rate [390]. These challenges mainly include mechanical stress in the blood, attack by the immune system, and anoikis [391]. In order to survive better in the circulatory system, tumour cells modulate the kinetics of focal adhesion by adaptation to enhance survival. Bioinformatics analyses have shown that focal adhesion proteins are usually more enriched in CTCs from colorectal cancer compared to primary tumour cells [392].

Both high and low stiffness tumour cells are well adapted to the complex dynamics of the circulatory system. This is because under mechanical stresses generated by blood flow, the dynamics and morphology of focal adhesions undergo adaptive regulation to resist these stresses and migrate. In order to resist damage to the plasma membrane from shear stresses generated by blood flow, CTCs tend to exhibit increased RhoA activity and strengthened F-actin filaments, which increase cellular stiffness [393]. At the same time, cells increase their adhesion levels and migration capacity in response to shear stress [394]. This has been demonstrated in a variety of tumour cells, such as breast cancer [394, 395], neuroblastoma [396] and hepatocellular carcinoma [397]. However, Xin et al. found that CTCs surviving under shear stress exhibited impaired the formation of focal adhesions and impaired F-actin, displaying low stiffness cellular characteristics [398]. This seemingly contradictory result may be related to the specific environment of the vascular system in which CTCs are located: for the most part, CTCs require enhanced F-actin to resist plasma membrane damage, whereas when passing through capillaries, CTCs require lower cellular stiffness in order to pass through narrow channels [399].

Anoikis is an apoptotic mechanism that occurs when cells lose adhesion to the ECM and usually results in non-malignant cell death [400]. The migration process of tumour cells often requires resistance to this apoptotic mechanism, as in the circulatory system, CTCs tend to have reduced contact with the ECM thus leading to insufficient information about the original matrix. In order to avoid this apoptosis, tumour cells mimic adhesion signals from the ECM through multiple pathways. *ZNF304* resists anoikis by transcribing integrin β1, which promotes the assembly of focal adhesions [401]. In addition, LRRC15 prevents ovarian cancer cells from undergoing loss-of-nest apoptosis by binding to integrin α5β1 and activating the FAK signalling pathway [402]. CTCs adapt to the environment of the vascular circulation and maintain their viability as much as possible by mimicking the “outside-in” signalling associated with the transmission of focal adhesions.

#### Extravasation

CTCs in the vascular system usually need to first arrest and extravasate into surrounding tissues to achieve effective metastasis, and their metastatic potential is dependent on extravasation capacity [391]. This process involves multiple steps, the primary condition being the ability of tumour cells to arrest and adhere to ECs.

Blood flow constitutes the first barrier to CTCs extravasation, and therefore the extravasation of tumour cells is significantly enhanced in the presence of blood stasis [403]. In this process, plasma fibronectin in the blood clot cross-links with FN, facilitating the stasis and adhesion of tumour cells to the endothelial ECM, which in turn drives extravasation [404]. In addition, FN deposits are often present on the luminal side of the hepatic vasculature, and these deposits enhance the interaction of focal adhesions with the endothelial ECM, leading to stagnation and promoting extravasation of CTCs, and ultimately liver metastasis [405]. Tumour cells are also able to bind directly to pre-exposed BM, thereby promoting lung metastasis [406]. This arrest and adhesion is also organ-specific and correlates with focal adhesion-ECM interactions. Taken together, focal adhesion-mediated attachment of CTCs to the endothelial ECM is a critical step in extravasation.

When CTC adheres to the endothelium, it needs to migrate across the endothelium in order to complete the transfer process. Similar to transendothelial migration during endocytosis, this also requires disruption of the endothelial barrier so that it can enter the tissue from the circulatory system. Pre-transfer matrix remodelling creates conditions similar to the tumour microenvironment, which destabilises the endothelial barrier. At the same time, dysregulation of focal adhesion dynamics does not only occur in tumour cells, but also in other cells of the malignant ECM [407]. Endothelial barrier function is closely related to the assembly of focal adhesions in ECs and its dysregulation (reviewed in [408]). Considering that dysregulation of focal adhesion dynamics in ECs weakens their barrier function, this would facilitate transendothelial migration of CTCs. At the same time, in order to break through the endothelial barrier, tumour cells form invadopodia structures, which enable extrusion from ECs junctions [409].

### Targeting focal adhesion in tumour metastasis

There are many key steps in the metastatic process that depend on the biological functions involved in focal adhesion. Therefore, how to exploit the mechanism of action of focal adhesion has become the focus of developing potential therapeutic targets. By targeting focal adhesion, researchers can effectively block the migration and invasion process of tumour cells, which provides new strategies and research directions for the development of anti-tumour metastatic drugs.

#### Targeting focal adhesion in tumour matrix remodelling

Among the most striking aspects of tumour matrix remodelling is tumour matrix fibrosis. Here, we take the example of tumour matrix fibrosis to illustrate how focal adhesion can be targeted in this process.

**Fig. 8:**
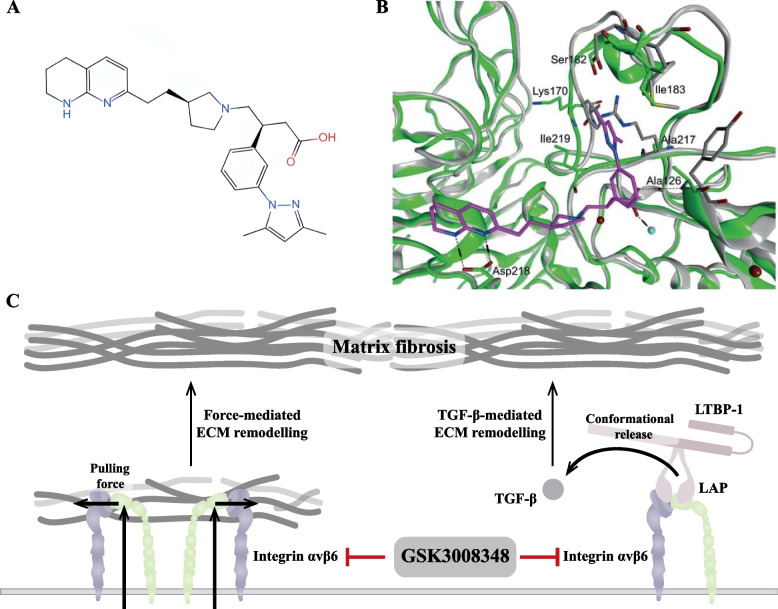
Schematic representation of the action of the integrin αvβ6 inhibitor GSK3008348. **A** Chemical structure of GSK3008348. Pictures drawn by https://indrawforweb.integle.com. **B** GSK3008348 acts as an RGD mimetic and interacts with integrin αvβ6 in a high-affinity manner dependent on key protein–ligand interactions, including amino acid residues Ser162, Lys170, and Ile183. Reprinted with permission from [412]. Copyright 2018 John Wiley and Sons. **C** GSK3008348 inhibits matrix fibrosis by inhibiting αvβ6 and interfering with its role in force-mediated ECM remodelling. Also, GSK3008348 inhibited TGF-β activation by interfering with αvβ6-induced conformational changes in LAP, thereby reducing TGF-β-mediated ECM remodelling and decreasing matrix fibrosis

Given the role of integrins in matrix remodelling mentioned above, it is reasonable to consider integrins as targets for tumour matrix anti-fibrosis. Currently, therapeutic strategies targeting integrins have been subjected to several clinical trials in the fibrotic process in a variety of diseases. For example, GSK3008348 (ClinicalTrials.gov: NCT03069989) and STX-100 (ClinicalTrials.gov: NCT01371305) targeting αvβ6 in the treatment of pulmonary fibrosis (Fig. 8). In addition, several reviews have described the use of integrins in tumour matrix-targeted therapy and matrix fibrosis treatment [266, 410, 411]. Although current strategies for targeting integrins have limited effectiveness in clinical tumour matrix therapy, it remains a promising target.

Targeting focal adhesion kinase components, such as FAK and Src, can prevent the process of tumour matrix fibrosis by inhibiting mechanotransduction at focal adhesion and thus inhibiting, for example, the phenotypic transformation of myofibroblasts and fibroblast migration [413]. The application of VS-4718 showed significant antifibrotic effects in a mouse model of pancreatic ductal adenocarcinoma [414]. Various Src inhibitors have also been used to treat the fibrotic process, such as Nintedanib, PP1, PP2 and SU-6656. Among them, the combination of Src inhibitor Dasatinib with EGFR inhibitors was able to inhibit the fibrotic process in pancreatic cancer tissues [415].

Other focal adhesion proteins have gradually entered the research horizon, and researchers have proposed a variety of potential research directions. For example, the N-terminal region of cav-1 contains caveolin scaffolding domain (CSD) which is considered to be an important site for its activity. Gauglitz et al. found that the process of TGF-β1-induced tissue fibrosis could be effectively reduced by the incorporation of CSD peptide [416]. In studies of obstructive nephropathy, QLT-0267 blocked TGF-β-induced LM-mediated fibrosis by inhibiting ILK [417]. Furthermore, in a mouse model of pressure overload, talin deficiency was shown to reduce fibrosis in the cardiac interstitium [418]. Although these studies have not been fully validated in tumours, they provide new directions for future anti-tumour fibrosis research.

#### Targeting focal adhesion in tumour cell migration and invasion

Numerous in vitro experiments have demonstrated that inhibition of focal adhesion protein function restricts the focal adhesion assembly, which in turn leads to failure of tumour cell migration and reduced tumour invasion [128, 419–425]. This inhibitory process is enabled by siRNAs as well as various focal adhesion protein inhibitors and has been demonstrated in several tumour types, including high-grade serous ovarian cancer [426], small cell lung cancer [427], epidermoid carcinoma [428], and others. Conversely, overexpression of focal adhesion proteins often leads to its increased assembly, promotes tumour cell migration and is associated with poor tumour prognosis [543]. However, T. Rodríguez-Rigueiro et al. showed that overexpression of Hakai decreases the levels of paxillin at cell–matrix adhesions and reduces the amount of focal adhesions, which in turn promotes the migration of tumour cells [429]. This phenomenon may seem to contradict the results that focal adhesion inhibition prevents tumour cell migration, but it actually reflects differences in the dynamics of focal adhesions at different sites.

During tumour cell migration, the dynamic balance between the assembly and disassembly of focal adhesions is critical for cell movement. Its assembly at the leading edge provides matrix anchoring for the cell, whereas its disassembly at the rear allows the cell body to contract and advance forward. It follows that the role of focal adhesion proteins in cell migration is not only localised but also spatially and temporally complex. The assembly and disassembly must be in dynamic balance during migration. This phenomenon suggests that the roles played by focal adhesion proteins at different locations are dynamic and complex, depending on the balance of the assembly and disassembly of focal adhesions. Meanwhiles, this phenomenon suggests that simply enhancing focal adhesion assembly of or promoting its disassembly may not be effective in inhibiting tumour cell migration. Excessive enhancement of the assembly of focal adhesions may lead to over-adhesion and impede cell movement, whereas over-promoting its disassembly may lead to loss of anchorage and ineffective cell advancement.

In order to effectively intervene in the migration of tumour cells, specific inhibition of the assembly or disassembly of focal adhesions in some specific regions has become an important research direction. By targeting the focal adhesion kinetic process at the leading or trailing edge and modulating the regional activity of focal adhesion proteins, it might be possible to inhibit the migration and invasion of tumour cells more effectively. This precise modulation needs to be further explored, especially in terms of how to design regional focal adhesion inhibitors or to influence the assembly and disassembly of specific focal adhesion regions through signalling pathways, which are potentially valuable for research.

### Focal adhesion proteins as therapeutic targets

Considering the critical role of focal adhesion in tumour development and metastasis, targeting focal adhesion could be an important approach for tumour intervention and therapy. Currently, interventions targeting focal adhesion components, including focal adhesion kinase components (e.g., FAK and Src), focal adhesion scaffolding proteins (e.g., ILK and talin), and integrins, and their phosphorylation activation are being extensively studied. Specific blockade or mutation of focal adhesion proteins significantly inhibits the assembly and function of focal adhesions. Therefore, targeting focal adhesion proteins is an effective strategy to inhibit the assembly and turnover of focal adhesions in tumour cells and provides a potential therapeutic avenue to inhibit tumour metastasis.

However, more studies are required to explore the potential risks of this strategy, especially because certain focal adhesion proteins (e.g., integrins) also have important functions at non-focal adhesion sites. Furthermore, FAK is involved in both the assembly of focal adhesions and the regulation of their turnover. Thus, blocking FAK may inhibit focal adhesion degradation, thereby enhancing their stability and promoting assembly [430]. Nonetheless, the function of focal adhesions is not limited to their assembly enhancement but also relies on their turnover during tumour cell migration. Therefore, targeted blockade against focal adhesion proteins still has potential as a specific strategy for tumour therapy. However, this raises an important question: does the blockade of focal adhesions affect the adhesion function of non-tumour cells? Addressing this issue needs thorough investigation into specific drug delivery approach to avoid adverse effects on normal cells in the future.

Table 2 lists the most recent clinical research status of targeted focal adhesion protein inhibitors as of 7 October 2024 (data from www.clinicaltrials.gov and http://www.chinadrugtrials.org.cn, covering the period from 2019 to 2024). The information provided in this table can help us to have a comprehensive overview of the latest research progress of currently targeted focal adhesion proteins, especially the developmental dynamics of drug combinations and therapeutic strategies, to provide a reference basis for further research and clinical application.

**Table 2:** Recent focal adhesion protein-targeting drugs in clinical studies (2021–2024)

Targeted component		Drug name	Drug type	Tumour type	Drug combination	Drug delivery	ClinicalTrials.gov ID	Time (First Posted)	Study status^a^		Results and publication		Study phase
Integrin	αvβ3	ProAgio	Novel proteins synthesized by computer simulation	Metastatic triple negative breast cancer	Gemcitabine	i.v	NCT06460298	2024/8/20	Recruiting		No results		Phase I/II
				Pancreatic ductal adenocarcinoma	Gemcitabine, Nab paclitaxel	i.v	NCT06182072	2023/9/14	Recruiting		No results		Phase I
				Advanced solid tumour malignancies		i.v	NCT05085548	2021/10/29	Recruiting		No results		Phase I
		BGC-0222	Small molecule	Advanced solid tumour malignancies		i.v	CTR20221496	2022/6/16	Recruiting		No results		Phase I
	α4β1	7HP-349 (Alintegimod)	Small molecule	Advanced solid tumour	Ipilimumab, Nivolumab	po	NCT06362369	2024/8/1	Recruiting		No results		Phase I/II
		CEND-1 (QLC12102)	Peptide	Pancreatic ductal adenocarcinoma	Gemcitabine, Nab paclitaxel	i.v	NCT06261359	2024/2/1	Recruiting		No results		Phase II
				Pancreatic cancer		i.v	NCT05052567	2021/10/21	Completed		Results available, not published		Phase I/II
				Pancreatic, colon and appendiceal cancers	Panitumumab, Folfirinox	i.v	NCT05121038	2021/10/20	Recruiting		No results		Phase I/II
		HYD-PEP06	Peptide	Advanced colorectal cancer		i.v	CTR20220769	2022/4/14	Not yet recruiting		No results		Phase II
				Solid tumour		i.v	CTR20213196	2022/1/25	Completed		Results available, not published		Phase I
	pan-αv	CEND-1 (QLC12102) ^b^											
FAK	FAK/Pyk2	Defactinib (VS-6063, PF-04554878)	Small molecule	Advanced lung adenocarcinoma	Avutometinib, Nivolumab	po	NCT06495125	2024/7/10	Recruiting		No results		Phase II
				Gastric cancer	Avutometinib	po	NCT06487221	2024/7/5	Recruiting		No results		Phase II
				Low grade serous ovarian cancer	Avutometinib, Letrozole	po	NCT06394804	2024/5/1	Recruiting		No results		Phase II
				Melanoma	Avutometinib, Encorafenib	po	NCT06194929	2024/1/8	Recruiting		No results		Phase I/II
				Metastatic cancer, pancreas cancer	Avutometinib, Gemcitabine, Nab-paclitaxel	po	NCT05669482	2022/12/30	Recruiting		No results		Phase I/II
				Chronic myelomonocytic leukaemia, acute myeloid leukaemia	Cedazuridine	po	NCT05636514	2022/12/5	Recruiting		No results		Phase I
				Endometrioid cancer, mucinous ovarian cancer, high grade serous ovarian cancer	Avutometinib	po	NCT05512208	2022/8/23	Recruiting		No results		Phase II
				Non-small cell lung cancer	Avutometinib, Sotorasib	po	NCT05074810	2021/10/12	Recruiting		No results		Phase I/II
		Conteltinib (CT-707)	Small molecule	Advanced pancreatic cancer	Gemcitabine	po	NCT05580445	2022/10/14	Recruiting		No results		Phase I/II
	FAK	IN10018 (BI 853520, Ifebemtinib)	Small molecule	Solid tumour	D1553	po	NCT06166836	2023/12/12	Recruiting		No results		Phase I/II
				Small cell lung cancer	Tislelizumab, Carboplatin, Etoposide	po	NCT06030258	2023/9/8	Recruiting		No results		Phase I/II
				Ovarian cancer	Pegylated liposomal doxorubicin	po	NCT06014528	2022/9/6	Recruiting		No results		Phase II
				Non-small cell lung cancer	Furmonertinib	po	NCT05994131	2023/8/16	Recruiting		No results		Phase I/II
				Locally advanced or metastatic solid tumour	Pegylated liposomal doxorubicin, Toripalimab	po	NCT05830539	2023/4/26	Active, not recruiting		No results		Phase I/II
				Pancreatic cancer	Albumin-bound paclitaxel, Gemcitabine, KN046	po	NCT05827796	2023/4/25	Recruiting		No results		Phase I/II
				Platinum-resistant ovarian cancer	Pegylated liposomal doxorubicin	po	NCT05551507	2022/9/22	Recruiting		No results		Phase I/II
				Solid tumour	D-1553	po	NCT05379946	2022/5/18	Recruiting		No results		Phase I/II
				Gastric cancer	Docetaxel	po	NCT05327231	2022/4/14	Recruiting		No results		Phase I
	FAK	Narmafortinib (AMP945)	Small molecule	Pancreatic cancer, pancreatic ductal adenocarcinoma, pancreatic ductal adenocarcinoma	Nab-paclitaxel, Gemcitabine	po	NCT05355298	2022/5/2	Completed		Results available and posted		Phase I/II
Src	Src/lck/Hck/c-Abl	Dasatinib	Small molecule	Triple-negative breast cancer	Quercetin, Taxane, Anthracycline, Eribulin mesylate, Vinorelbine, Capecitabine, Carboplatin, UTD1, Platinum	po	NCT06355037	2024/4/9	Recruiting		No results		Phase II
				Head and neck squamous cell carcinomas	Tislelizumab, Quercetin	po	NCT05724329	2023/2/13	Recruiting		No results		Phase II
				Chronic myeloid leukaemia	Ketoconazole	po	NCT05638763	2022/12/6	Recruiting		No results		Phase II
	Src/Abl	Bosutinib	Small molecule	Chronic myeloid leukaemia, chronic myelogenous leukaemia	Olverembatinib	po	NCT06423911	2024/5/21	Recruiting		No results		Phase III
	Src/Abl	Ponatinib	Small molecule	Leukaemia	3L Therapy	po	NCT05743465	2023/2/24	Completed		No results		N.A
				Chronic myeloid leukaemia	Imatinib, Nilotinib, Bosutinib	po	NCT05286528	2022/3/18	Completed		Results available and posted		N.A
	Src/Yes1	NXP-900	Small molecule	Advanced solid tumour		po	NCT05873686	2023/5/24	Recruiting		No results		Phase I
	Src	Tirbanibulin (KX2-391)	peptide mimetic	Basal cell carcinoma	Tirbanibulin	topically apply	NCT06112522	2023/11/1	Recruiting		No results		Phase II
				Superficial basal cell carcinoma	Tirbanibulin ointment	topically apply	NCT05713760	2023/2/6	Completed		Results available, not published		Phase II

Emerging focal adhesion protein-targeted therapies currently in clinical trials or emerging focal adhesion protein-targeted therapies that are in clinical trials but have not yet published any study results. This table, selected from data from 2021–2024, details therapeutic novel potential drugs targeting focal adhesion proteins. Data were queried at http://www.clinicaltrials.gov and http://www.chinadrugtrials.org.cn. The date of access is October 7, 2024.* i.v.* intravenous, *po* peros

^a^Excluded Not yet recruiting, Withdrawn, Terminated

^b^Multiple occurrences, presented only the first time

Table 3 lists the current inhibitors targeting focal adhesion proteins that have reported clinical safety and efficacy. This table helps us to better understand the key results of these inhibitors in clinical trials, thus providing an important reference for future research and drug development. The table also provide key data on the prospects for the clinical application of antitumour therapies targeting focal adhesion proteins. Moreover, a search for successfully developed and marketed inhibitors targeting focal adhesion proteins revealed that, while inhibitors of FAK, Src, and integrins are available on the market in small numbers, the number of inhibitors targeting other focal adhesion proteins remains relatively low. Most of these inhibitors are directed at the treatment of non-tumour diseases, underscoring the necessity for further in-depth research in the area of drug development for focal adhesion proteins that targets tumour-related therapies which has a large potential.

**Table 3:** Current inhibitors targeting focal adhesion proteins with reported clinical safety and efficacy

Targeted component		Drug name	Drug type	Drug delivery	Tumour type	Drug / interventions combination	ClinicalTrials.gov ID	Total number of patients	Study assessment indicators	Study design and treatment	Safety and efficacy
Integrin	pan-αv	Intetumumab	Humanized mAb	i.v	Melanoma	Dacarbazine	NCT00246012	129	PFS; OS, ORR, adverse events, pharmacokinetics	10 mg/kg, q3w	Favourable safety profile, no efficacy [431]
					Prostate cancer	Docetaxel, Prednisone	NCT00537381	131	PFS; CR, PR, PSA, OS	10 mg/kg, q3w	All efficacy end-points favoured [432]
		Abituzumab (DI17E6, EMD 525797)	Humanized mAb	i.v	Colorectal cancer	Cetuximab, Irinotecan	NCT01008475	216	PFS; OS, RR, tolerability	1000 mg, q2w	Acceptable tolerability, no efficacy [433]
					Prostate cancer	LHRH agonist	NCT01360840	180	PFS	1500 mg, q3w	Specific activity [434]
					Prostate cancer		NCT00958477	26	TEAEs, DLTs	1500 mg, q2w	Acceptable safety, limited effectiveness [435]
		GLPG0187	Small molecule	Continuous i.v. infusion	Glioma, solid tumours		NCT01313598	20	PK	400 mg/d	Favourable toxicity profile, no efficacy [436]
		MK-0429	Small molecule	po	Prostate cancer and bone metastases		NCT00302471	21	Biochemical markers, bone turnover	1600 mg, b.i.d	Well tolerated [437]
	αvβ3, αvβ5	Cilengitide (EMD 121974)	Peptide	i.v	Glioblastoma	Temozolomide chemoradiotherapy	NCT00689221	3471	OS	2000 mg, q2w	No efficacy [438]
					NSCLC	Cetuximab, chemotherapy	NCT00842712	169	PFS; OS, safety, biomarker analyses	2000 mg, b.i.w	Potential clinical activity [439]
	αvβ3	etaracizumab (Abegrin)	Humanized mAb	i.v	Advanced solid tumours		NCT00263783	16	PK	6 mg/kg, q.w	Well tolerated [440]
	α5β1	MINT1526A (RG-7594)	Humanized mAb	i.v	Advanced solid tumours	Bevacizumab	NCT01139723	54	Safety assessments	30 mg/kg, q3w	Well tolerated, limited effectiveness [441]
	β1	ATN-161	Peptide	i.v	Solid tumours		N.A	26	Toxicity assessment, tumour burden	16 mg/ kg, t.i.w	Well tolerated [442]
FAK	FAK/Pyk2	Defactinib (VS-6063, PF-04554878)	Small molecule	po	NSCLC		NCT01951690	75	PFS; ORR, OS, safety, tumour biomarkers	400 mg, b.i.d	Well tolerated, modest clinical activity [443]
		PF-00562271	Small molecule	po	Advanced solid tumours		NCT00666926	99	Safety, tumour response, PK, PD	125 mg, b.i.d	Tolerable [444]
	FAK	IN10018 (BI 853520, Ifebemtinib)	Small molecule	po	Metastatic solid tumours		NCT01905111	21	MTD; PK, CR, PR, SD	200 mg/d	Acceptable safety profile, favourable pharmacokinetics, and potential anti-tumour activity [445]
		GSK2256098	Small molecule	po	Glioblastoma		NCT01138033	13	MTD; PK, antitumor activity	1000 mg, t.i.w	Tolerable [446]
Src	Src/Lck/Hck/Abl	Dasatinib	Small molecule	po	Metastatic colorectal cancer	Modified FOLFOX6, Cetuximab	NCT00501410	77	MTD, DLT, response-rate distribution; OS, safety profile and tolerability	200 mg/d	Modest clinical activity [447]
	Src/Bcr-abl	Bosutinib	Small molecule	po	Glioblastoma		NCT01331291	16	PFS; ORR	400 mg/d	No efficacy [448]
		Saracatanib (AZD0530)	Small molecule	po	Metastatic melanoma		NCT00669019	23	ORR; PFS	175 mg/d	Limited efficacy [449]
		AZD0424	Small molecule	po	Advanced solid tumours		NCT01668550	41	MTD, DLT	150 mg/d	No pharmacodynamic effect [450]
	Src	KX2-391	Small molecule	po	Solid tumours		NCT00658970	44	MTD, DLT	40 mg, b.i.d	Favourable pharmacokinetic profile, well tolerated [451]

Studies that have published safety, tolerability or efficacy data are included. *i.v.* intravenous, *PFS* progression-free-survival, *OS* overall survival, *ORR* objective response rate, *CR* complete response, *PR* partial response, *PSA* prostate-specific antigen, *TEAEs* treatment-emergent adverse events, *DLTs* dose-limiting toxicities, *PK* pharmacokinetics, *po* peros, *SD* stable disease, *MTD* maximum tolerated dose, *PD* pharmacodynamics

#### Integrin inhibitors

The activation and aggregation of integrins, which are core components directly connected to the ECM, are early events in the assembly of focal adhesions, making integrin inactivation a potential strategy in tumour intervention. Antagonising integrins usually reduces their mediated cell adhesion and migration, thereby inhibiting focal adhesion assembly and downstream signalling [47, 424, 425].

Most integrin inhibitors block integrin downstream signalling by competitively binding to their endogenous ligand-binding sites; such inhibitors are known as competitive inhibitors [452]. These inhibitors often contain sequences related to the target integrin ligand, such as RGD, KGD, IDS, or LDV sequences. By mimicking the competitive binding of endogenous ligands to integrin binding sites, they can effectively reduce integrin-mediated adhesion and migration with high specificity. For example, Cilengitide (EMD 121974) has a high binding affinity for αvβ3 and αvβ5 integrins (IC_50_ = 30–50 nM) and low effect on platelet glycoprotein [GP] IIb/IIIa adhesion. However, paradoxical activation effects of ligand mimetics have also been reported, which have led to the failure of clinical trials and unanticipated adverse drug reactions. This issue has been found in several integrin inhibitors targeting αIIbβ3 [453] and αvβ3 [454, 455], among others. For example, low concentrations of Cilengitide unexpectedly promoted tumour growth and angiogenesis in vivo, contrasting with the findings from in vitro experiments [454, 455]. This unexpected outcome behind the failure of the clinical trials of Cilengitide failed. To address such issues, drug combinations, such as Cilengitide and Verapamil combination, have been proposed to enhance the efficacy of Gem [456]. Even at low doses, Cilengitide promotes angiogenic activity in vivo, rather than functioning as the originally expected antiangiogenic therapy.

The focal adhesion assembly is dependent on integrin conformational changes, which bring about integrin binding to ECM ligands and “outside-in” signalling. Compounds that inhibit the activation of integrins by blocking their orthosteric binding site are known as allosteric inhibitors [452]. Research on this type of inhibitors, mainly focusing on leukocyte integrins, with only limited studies on variant inhibitors of focal adhesion-integrins (e.g., αv [457], αvβ3 [458, 459], α4β1 [460], and α2β1 [461]). Most of them are in the preclinical stage. The structural design of variant inhibitors can effectively address integrin agonism triggered by competitive inhibitors. Competitive inhibitors, such as drugs containing RGD sequences, usually bind to their ligand binding sites by inducing a conformational change in the integrin. However, this conformational change introduces a potential integrin agonist effect that can lead to harmful side effects. To avoid this problem, researchers have structurally designed molecules to replace the Ser residue after the RGD sequence with Trp, which is able to form a π-π interaction with Tyr122 of the β3 chain, thus preventing the conformational change of the β subunit [462]. Additionally, the NGR sequence generates isoDGR upon asparagine deamidation, which binds to the RGD binding site via an inverted orientation [463]. The isoDGR-based cyclic peptide CisoDGRC maintains high affinity and does not induce conformational changes in integrins, making it a pure αvβ3 receptor inhibitor [459]. Other pure integrin inhibitors have been developed, showing promising results at low doses [464].

Although variant inhibitors are able to radically inhibit integrins and solve the integrin agonism problem described above, however, they also face problems such as systemic toxicity [465]. Since these inhibitors often inhibit multiple integrins simultaneously, they are prone to unexpected "off-target effects” and toxicities. An off-target effect is the unintended binding of a drug to an unintended target. This binding may be neutral and produce no noticeable effects, but it may also lead to unintended toxic effects. Abituzumab (DI17E6, EMD 525797) is able to bind not only to the orthosteric binding sites of integrins, but also to the extracellular structural domains of the Integrin αv chain, thereby inhibiting the activation of multiple integrins [457]. Its pan-inhibitory profile against multiple integrins has shown some efficacy in clinical trials, but toxicity events have been reported from time to time [433–435].

Despite the key role of integrins in cell adhesion, migration, invasion and angiogenesis observed in a wide range of tumours, the overall performance of integrin-based inhibitors in oncology clinical trials has fallen far short of expectations. This may be related to the dual or multiple roles of integrins in tumour biology. For example, Reader et al. effectively inhibited the growth of pancreatic ductal adenocarcinoma in mice by combination treatment with the αvβ6 antibody 264RAD and gemcitabine [466]. However, Sipos et al. found that blocking αvβ6 did not show an inhibitory effect on tumour sphere formation in pancreatic ductal adenocarcinoma through an in vivo study [467]. Similarly, E7820 (α2 integrin inhibitor) was once considered to have broad anti-tumour potential due to its ability to inhibit tumour angiogenesis [468]; however, clinical trials in advanced solid tumours have only shown limited efficacy [469]. Cilengitide (αvβ3 and αvβ5 integrin inhibitor) has demonstrated good efficacy in a mouse tumour model [470] and in vitro experiments with glioma cells [471] demonstrated good pharmacodynamic effects, but failed to achieve the expected therapeutic effects in several clinical trials involving cancers such as glioblastoma [438] and non-small cell lung cancer [439] (ClinicalTrials.gov: NCT00689221, NCT00842712), and its further development as a tumour therapeutic agent is, therefore, hampered. Lack of sufficient specificity, potential agonistic effects, and the complex tumour microenvironment are all potential reasons for clinical trial failure or minimal efficacy.

Although integrin inhibitors have shown therapeutic promise in other diseases (e.g., ulcerative colitis, Crohn's disease, etc.) [472], most of the integrins targeted in these diseases are αLβ2, α4β7, and αIIbβ3, rather than αvβ3, αvβ5, and α5β1, which are the main ones contained in focal adhesion. Due to the large number of integrins, their multiple functions and the complexity of the tumour microenvironment, a more systematic assessment of the safety and specificity of the drugs and their interactions with other signalling pathways is required during clinical translation. Thankfully, several clinical studies have reported the controlled safety of certain focal adhesion-targeted integrin inhibitors [433, 436, 438, 473], which provides a viable entry point for their further exploration in tumour therapy. In recent years, in order to increase therapeutic efficiency and reduce toxicities and off-target effects, researchers have attempted to employ a variety of strategies, including the use of nano-delivery systems to improve integrin-targeted drug delivery [474, 475] and the design of highly selective drugs for specific integrin subtypes, such as Peptide-drug conjugates [476], Antibody–drug conjugates [477], and proteolysis targeting chimera (PROTAC) [478].The continuous improvement of these strategies provides more feasibility and potential for the clinical application of integrin-targeted therapy in tumours.

#### FAK inhibitors

FAK is one of the most important protein kinases in the assembly and turnover of focal adhesions. It acts by phosphorylating a variety of focal adhesion proteins, and its inhibition usually alters the kinetics of focal adhesion, which in turn inhibits the migratory ability of tumour cells [419, 420, 423]. As FAK is overexpressed in a variety of tumours, a series of FAK inhibitors have been developed and used in clinical studies in recent years, including GSK2256098, Defactinib (VS-6063, PF-04554878), VS-6062 (PF 562271), VS-4718 (PND-1186), and IN10018 (BI 853520, Ifebemtinib) [479–481]. The development of FAK inhibitors has focussed on two main areas: kinase-dependent inhibition and kinase-independent inhibition. The former include ATP-competitive kinase inhibitors and alternative kinase inhibitors, while the latter act by inhibiting the scaffolding function of FAK, specifically including FAK-FERM inhibitors and FAK-FAT structural domain inhibitors [482]. Recently, new FAK inhibitors have been designed and developed owing to the development of PROTAC technology [483]. Several studies have shown that FAK inhibitors significantly inhibit the formation and turnover of focal adhesions in tumour cells [484–487]. However, FAK inhibitors still face multiple challenges in clinical application, such as selectivity and toxicity, and require further in-depth research.

Currently, most FAK inhibitors are ATP-competitive kinase inhibitors with unique chemical structures that allow them to specifically target the ATP binding site of FAK. These inhibitors typically contain pyrimidine and pyridine chemical groups that bind to the hinge region of the ATP binding site, termed ATP site hinge binders. Due to the highly conserved nature of the ATP binding site in tyrosine kinases, these FAK inhibitors often require additional restrictions to achieve selectivity for FAK. FAK shares a similar catalytic kinase structural domain with Pyk2, and therefore most aminopyrimidine analogues (e.g., VS-6062) inhibit both kinases [488]. By replacing the oxyindole ring in VS-6062 with a phenyl group, its binding activity to FAK was significantly reduced due to the inability of the phenyl ring to form a hydrogen bond with R426 in FAK [489]. By replacing the solvent-exposed site of VS-6062 with a benzenesulfonamide moiety, PF-431396 was obtained; this exhibited greater potency and longer residence time in binding to FAK compared to its predecessor [489].

TAE226 (NVP-226) can inhibit FAK by inducing a unique change in the DFG conformation of FAK, which is different from that of other tyrosine kinases, thereby improving its selectivity and inhibition efficiency [490]. Additionally, the presence of the highly conserved glycine residue Gly563 before the DFG motif in FAK, a unique structure, further enhances the selectivity of FAK inhibitors [490]. Overall, the selectivity of FAK inhibitors is dependent on the stability of the helical conformation upon binding to the DFG motif, the tight binding of the inhibitor conformation, and the formation of hydrogen bonds between FAK and the inhibitor. IN10018 stands out as a highly selective FAK inhibitor, with its IC50 value measured by DELFIA showing a strong selectivity difference between FAK and Pyk2 (FAK: IC_50_ = 1 nM, Pyk2: IC_50_ > 50,000 nM) [491]. In patients with advanced or metastatic malignancies, IN10018 showed good tolerability (ClinicalTrials.gov: NCT01335269, NCT01905111) [445, 492]. In addition, IN10018, in combination with pegylated liposome doxorubicin/doxorubicin, was able to enhance immunogenic cell death, thus demonstrating enhanced anti-tumour activity [493]. Nevertheless, off-target effects of FAK kinase-dependent inhibitors still require further attention and research [494, 495].

Nevertheless, there are some problems with FAK inhibitors based on ATP-competitive kinase inhibitor design. Although the selectivity of ATP-competitive inhibition can be improved by pre-drug design via phenyl rings, some FAK inhibitors still accidentally inhibit Pyk2, Src or other kinases, leading to additional toxic side effects or pharmacodynamic uncertainties, as ATP-binding sites are also widely present in other tyrosine kinases [494, 495]. In addition, when tumour cells are exposed to ATP-competitive inhibitors for a prolonged period of time, adaptive changes in the kinase structure or pathway may occur, which in turn reduces the long-lasting efficacy of the drug [496]. Meanwhile, FAK is not only highly expressed in tumour cells, but also plays a role in normal cell adhesion, survival and immune regulation. Therefore, the risk between high dose and potential side effects needs to be balanced clinically.

To further reduce off-target effects, some studies have used alternative kinase inhibition pathways, whereby inhibition is achieved by binding to atypical kinase regions of FAK rather than the traditional ATP site [482]. These types of inhibitors are expected to improve selectivity, but are currently in preclinical or early clinical studies with relatively limited clinical data. The FAK inhibitory effects of various chemical scaffolds have been intensively investigated, and exploring these chemical scaffolds can help to develop novel FAK inhibitors, such as diphenylpyrimidines, triazines and pyrrolopyrimidines [481]. Among them, pyrimidine, particularly diphenylpyrimidine, scaffolds are the most studied chemical scaffolds. Defactinib, a FAK inhibitor, is based on this scaffold. Of these, pyrimidine (especially diphenylpyrimidine) scaffolds are the most widely studied structures, and defactinib is the FAK inhibitor based on this scaffold. Defactinib is a second-generation FAK inhibitor targeting FAK and Pyk2, and preclinical studies have demonstrated excellent inhibitory potency (IC_50_ = 0.6 nM) (ClinicalTrials.gov: NCT00787033) [497]. In a phase II clinical trial for advanced KRAS-mutated non-small cell lung cancer, defactinib monotherapy demonstrated modest clinical activity [443]. In addition, defactinib, in combination with pembrolizumab and gemcitabine, demonstrated significant remission in the treatment of advanced pancreatic cancer (ClinicalTrials.gov: NCT02546531) [498]. Another FAK inhibitor based on the same scaffold is TAE226, which has an IC50 of 5.5 nM and can exert significant anti-tumour activity in non-small cell lung cancer carrying EGFR mutations, including the T790M mutation [499]. In addition, TAE226, when combined with Sorafenib, has shown more significant anti-tumour effects in hepatocellular carcinoma compared to other FAK inhibitors (e.g., PND1186 and PF431396) [500]. However, TAE226 has not entered clinical trials due to its effect on glucose metabolism.

Kinase-independent inhibitors’ function by disrupting the FAK scaffold protein–protein interactions and can be classified into FAK-FERM structural domain inhibitors and FAK-FAT structural domain inhibitors according to their site of action. Specifically, these two classes of inhibitors prevent FAK activation by inhibiting phosphorylation at different sites of FAK, such as FAK-FERM structural domain inhibitors targeting Tyr397 and FAK-FAT structural domain inhibitors targeting Tyr925 [501]. Compared to kinase-dependent inhibitors, kinase-independent inhibitors tend to be more selective by inhibiting FAK-specific protein–protein interaction sites, reducing the unintended interference with other kinases, and are therefore increasingly valued by researchers. Meanwhile, many ATP-competitive inhibitors are prone to produce active metabolites when metabolised in vivo, which may lead to liver and kidney toxicity, and non-dependent inhibitors can effectively reduce this clinical problem [502]. Through computerised molecular docking techniques, a variety of potential kinase non-dependent inhibitors have been identified and developed, such as the FAK-FERM structural domain inhibitor 1,2,4,5-benzenetetraamine tetrahydrochloride [503], 1-(2-hydroxyethyl)−3,5,7-triaza-1-azoniatricyclo[3.3.1.1(3,7)]decane (Y11) [504], 16-hydroxy-cleroda-3,13-dien-16,15-olide (HSD) [505] and the FAK-FAT structural domain inhibitor chloropyramine hydrochloride (C4) [506], among others. In addition, the antiproliferative activity of existing inhibitors can be significantly improved by structural optimisation, such as replacing the chlorine group in C4 with trifluoromethoxy or tert-butyl groups (IC50 = 4.59–5.28 μM) [484].

Due to the complex functions of FAK in cytoskeletal remodelling, cell migration and regulation of the immune microenvironment, these inhibitors still need to be systematically evaluated for safety and efficacy in preclinical and clinical trials. Currently, most kinase non-dependent inhibitors are still in the early stage of research, and relatively limited varieties have actually entered the clinic. For example, GSK2256098, classified as a FAK-FERM structural domain inhibitor by inhibiting autophosphorylation at the FAK Tyr397 site, has demonstrated good tolerability and efficacy in patients with recurrent merlin-negative mesothelioma and recurrent/progressive NF2-mutant meningiomas (ClinicalTrials.gov: NCT01138033, NCT02523014) [507–509]. However, more clinical data are still needed to validate the assessment of its specific mechanism of action and potential side effects.

Overall, the clinical efficacy of existing FAK inhibitors still fails to meet expectations, and how to ensure high selectivity while reducing off-target effects and toxicities, and making full use of the tumour microenvironment and immune regulatory mechanisms, remains a key issue in the development of this field. The safety and efficacy of AAK inhibitors need to be evaluated through larger and more rigorous clinical trials. In addition, combination strategies [167, 510], the application of novel drug delivery systems [511, 512], and the development of targeted degradation agents based on PROTAC technology [513–515] are expected to further improve the therapeutic selectivity, reduce the toxicity and side-effects, and solve the problem of partial resistance, thus promoting the maximisation of the potential of FAK inhibitors in oncology treatment.

#### Src inhibitors

Src inhibitors appear to be more established and clinically reliable in tumour therapy compared to integrin inhibitors and FAK inhibitors. Several Src inhibitors such as Bosutinib, Dasatinib, Ponatinib and Vandetanib have been approved by the FDA and play important roles in the treatment of a variety of malignant tumours [516]. Src is recruited to focal adhesion by FAK to form a kinase complex with FAK, phosphorylating a variety of focal adhesion proteins and thus regulating focal adhesion assembly [141]. Like FAK, the phosphorylation function of Src is critical for the dynamics of focal adhesion. Numerous studies have shown that mutations in Src in cells often lead to failure of the assembly and disassembly of focal adhesions [287, 430, 517–520]. Because of this key role, Src inhibitors are seen as potentially important components in focal adhesion-targeted therapy.

Src is a member of the Src family kinases (SFKs) which acts by phosphorylating a variety of target proteins. Most Src inhibitors currently in clinical use are multi-target kinase inhibitors that act mainly by reversibly competing for ATP binding sites. For example, Bosutinib has inhibitory activity against all SFKs and Abl kinases; Saracatinib is likewise an inhibitor of Src and Abl kinases; and Dasatinib has a broad spectrum of inhibitory activity against a wide range of SFKs, including Src, Lck, Fyn and Yes (reviewed in [516]). The high degree of structural homology between Src and members of other SFKs, especially those sharing similar ATP-binding sites, makes the development of specific Src inhibitors a great challenge [521, 522]. To overcome this challenge, Chakraborty et al. designed an inhibitor that specifically targets Lyn by replacing the non-selective chemical scaffold with a conformationally flexible helix αC [523]. This strategy provides new ideas for designing Src-specific inhibitors. In recent years, some compounds specifically targeting Src, such as PP1 [524] and PP2 [525], have been developed. However, these Src inhibitors have only been used in animal model experiments at present and have not entered clinical trials.

Pan-Src inhibitors, represented by Bosutinib, are mainly used for the treatment of haematological tumours. Bosutinib and Dasatinib have been approved by the FDA as first-line treatment for Philadelphia chromosome-positive chronic-phase chronic myeloid leukaemia (CML) [526, 527]. Recently, Bosutinib has also been approved for the treatment of paediatric patients with CML, further demonstrating its favourable clinical safety profile [528]. In addition, ponatinib has been approved for the treatment of patients with the BCR-ABL1^T315I^ mutation [529], which highlights its therapeutic benefits in the context of specific genetic mutations. In addition, these drugs are actively undergoing clinical trials involving cancer types other than haematological tumours. For example, in a randomised, double-blind phase III clinical trial (ClinicalTrials.gov: NCT00410761), Vandetanib showed therapeutic efficacy in medullary thyroid carcinoma [530]. In combination with docetaxel, Vandetanib significantly prolonged progression-free survival after first-line treatment in patients with advanced non-small cell lung cancer [531]. However, Bosutinib and Dasatinib have not performed as well in breast cancer. A phase II clinical trial (ClinicalTrials.gov: NCT00880009) showed limited therapeutic efficacy of Bosutinib in breast cancer [532], and Dasatinib monotherapy for triple-negative breast cancer had similarly limited results [533–535]. This suggests that the indications for pan-Src inhibitors vary markedly in different tumour types, and that more optimal results may need to be achieved through combination therapy or with other signalling pathway-targeting agents.

In addition, several non-ATP-competitive Src inhibitors exist, one of which is KX2-391, which exhibits high selectivity for Src by targeting the enzyme-matrix binding domain rather than the ATP binding site. As an orally active Src inhibitor, KX2-391 effectively inhibits Src-catalysed phosphorylation of focal adhesion proteins such as FAK and paxillin [451]. In a phase Ib study (ClinicalTrials.gov: NCT01397799) in elderly acute myeloid leukaemia, KX2-391 showed good tolerability with a maximal tolerated dose (MTD) of 120 mg per day and exhibited anti-tumour biological activity [536]. In another phase I study (ClinicalTrials.gov: NCT00658970) in advanced malignant tumours, KX2-391 was administered orally twice daily at an MTD of 40 mg and showed a good pharmacokinetic profile and tolerability [451]. In addition, the same type of Src inhibitor includes KX01, and these compounds show potential in cancer therapy, but their long-term safety, off-target effects, and optimal dosing regimen need to be further validated.

Compared with integrin inhibitors and FAK inhibitors, Src inhibitors have accumulated richer clinical experience and a certain degree of success in oncology treatment, especially in haematological tumours, where they have demonstrated good safety and efficacy [537]. However, they also face challenges such as off-target effects, resistance mechanisms, and indication limitations, leading to their limited efficacy in the monotherapy of certain solid tumours [537–539]. In order to increase the clinical value of Src inhibitors in tumour therapy, more specific inhibitors can be designed through structural biology and conformational relationship studies in the future [540, 541], and efficient combination regimens and other therapeutic strategies can be explored [542]. In addition, individualised therapy and biomarker screening could help identify patient groups that are more sensitive to Src inhibitors, thereby improving the efficacy and safety of treatment [543–545].

#### Advances in other focal adhesion protein inhibitors

Compared to the aforementioned studies targeting focal adhesion proteins, the development of ILK, talin and cav-1 inhibitors appears to be scarce. However, due to the unique localisation and roles of ILK, talin and cav-1 in focal adhesion, these three classes of inhibitors may be potentially more important than others in regulating FA kinetics. Despite the relatively limited research on these inhibitors, their unique functions make targeting these proteins likely to have a more significant impact on inhibiting the assembly and turnover of focal adhesions, and even migration and invasion in tumour cells. Therefore, the development of inhibitors targeting ILK, talin and cav-1 will provide new breakthroughs in the regulation of focal adhesion dynamics and related tumour therapies.

ILK was originally thought to be a serine/threonine kinase involved in regulating the cytoskeleton and related proteins by phosphorylating focal adhesion proteins, thereby exerting signalling and cell motility functions [546]. However, in recent years, it has been shown that ILK is actually a pseudokinase, whose pseudo active site conformation does not possess conventional kinase activity, but mainly acts as a mechanically linked scaffold that interacts with proteins such as parvin to maintain cytoskeletal homeostasis [547, 548]. For example, QLT-0267 was once considered a kinase inhibitor of ILK, capable of inhibiting ILK and FAK functions and playing a role in LN-induced matrix deposition [549]. Meanwhile, in combination with Imatinib (Abelson kinase inhibitor), QLT-0267 is effective in inhibiting aberrant proliferation in glioblastoma [550]. However, given the pseudokinase nature of ILK, the specific mechanism of action of QLT-0267 needs to be further explored. Its inhibitory effect might stem more from off-target effects, such as inhibition of FAK or other key signalling molecules. Another ILK inhibitor designed based on structural optimisation, N-methyl-3-(1-(4-(piperazin-1-yl)phenyl)−5-(4′-(trifluoromethyl)-[1,1′-biphenyl]−4-yl)−1H-pyrazol-3-yl)propanamide was reported to reduce the expression of YB-1, HER2 and EGFR via ILK-dependent reduction, and no inhibitory effects on other kinases were observed, showing high inhibitory specificity [551]. This suggests that the development of highly specific ILK inhibitors is feasible, but systematic pharmacokinetic and toxicological studies of this class of molecules are lacking and entry into clinical trials has not been reported.

In view of the protein scaffolding role of ILK, researchers have begun to explore the development of more specific ILK inhibitors by inhibiting its scaffolding function. Garcia-Marin et al. used computer simulation to design a series of tripeptide molecules based on the α-parvin fragment to affect ILK-dependent phenotypic regulation by inhibiting ILK-parvin interactions, which offers a new idea for the future development of ILK inhibitors [552]. Although these studies provide an important basis for the development of ILK inhibitors, no clinical trials have been conducted to further validate the effects of ILK inhibitors, suggesting that research in this area is still at an early stage.

Compared to targets such as FAK and Src, which have been clinically tested in a wide range of tumours, ILK inhibitors are still in the laboratory or preclinical research stage, and there is a lack of data from large-scale clinical trials to fully assess their safety and efficacy. In addition, ILK inhibitors may face the risk of off-target effects and toxicities, which are closely related to their adhesion and signal regulation functions in normal tissues. Therefore, in future studies, dose balancing and precise administration will be the key aspects to ensure the success of clinical translation of ILK inhibitors.

Talin plays a "scaffolding core" role in focal adhesion: conformational changes between its head and rod structure determine integrin activation and focal adhesion assembly [553]. Talin targeting strategies are also relatively limited compared to ILK, but because talin is pivotal in the stabilisation and disassembly of adhesion complexes, inhibition of talin may interfere more directly with focal adhesion dynamics [554, 555]. Considering the unique structure of talin, talin inhibitors act mainly by blocking the interaction between the talin head and the rod structure, as well as inhibiting the interaction of integrins with talin. When TA205 was microinjected into cells, the structure of focal adhesion was significantly disrupted [556]. The disruption occurs because TA205 induces metastable inhibition of talin by inhibiting talin head-rod interactions, which further leads to the disassembly of focal adhesions [556]. In addition, Gao et al. designed an experimental peptidomimetic inhibitor, S-TBS, which has a stronger binding force and can effectively inhibit the interaction of talin with integrins, thus preventing talin-mediated integrin activation [557]. This inhibitor not only targets the direct binding of talin to integrins but also inhibits the translocation and activation of talin by blocking the interaction between the talin rods and the PM-anchored RIAM, realising a “double-strike” strategy [557]. S-TBS has shown good cell permeability in experiments with minimal cytotoxicity, showing great promise for application [557]. However, although these inhibitors have shown encouraging results in cellular and animal models, their potential in tumour therapy still needs to be validated by further clinical trials.

Unlike other molecules, talin has a large molecular weight and complex functional domain interactions, and it remains difficult to accurately interfere with its head-rod or talin-integrin site of action in vivo without affecting other key cellular functions. At the same time, talin has a wide range of potential effects and plays an indispensable role in various physiological functions, such as normal cell adhesion, immune cell migration and even platelet aggregation. Excessive inhibition of talin may have adverse consequences such as immunodeficiency, thrombosis or bleeding risk [558, 559].

Several plant-derived compounds, such as daidzein, salidroside and curcumin, are commonly used as cav-1 inhibitors [560–563]. However, these compounds often lack specificity in their targeting. The caveolin-1 CSD, an amino acid sequence located at positions 82–101, is thought to play an important role in the regulation of caveolae client proteins [564]. Exogenous peptides containing the CSD can inhibit cav-1 activity by competing with endogenous cav-1 for binding to downstream cellular signals. For instance, CSD peptides inhibit fibrotic processes both in vivo and ex vivo [565]. The novel CSD peptide LTI-03, a seven-amino-acid peptide derived from CSD, is being developed for the treatment of idiopathic pulmonary fibrosis (ClinicalTrials.gov NCT04233814, NCT05954988). In addition, Gilliam et al. designed WL47 by optimizing the T20-derived cav ligand, which binds with high affinity to cav-1, thereby disrupting cav-1 function and its associated pathways [566].

#### Current issues in targeting focal adhesion proteins

In conclusion, focal adhesion plays an important role in tumourigenesis, progression, and metastasis. Existing cellular experiments and animal models have verified that targeting focal adhesion can inhibit tumour metastasis. However, there are no clinical trials using focal adhesion status as an observational indicator. The possible reason for this lies in its complexity in efficacy assessment. Focal adhesion is highly dynamic in tumour cells and even differs between anterior and posterior poles of the same cell. Therefore, the observation of focal adhesion characteristics may not be as intuitive or easy to quantify as existing clinical trial metrics.

With the rapid development of deep learning technology, using it for automated analysis of focal adhesion features has become a feasible direction. Currently, deep learning has been widely used in biological research fields, such as complex pattern recognition [567, 568] and bio-optical detection [569]. Deep learning is capable of recognising complex feature patterns efficiently and has the ability to handle massive data. Therefore, applying deep learning techniques to the analysis of focal adhesion features not only overcomes the limitations of traditional means, but also provides new possibilities for in-depth study of the dynamics of FA in tumours, which is an emerging field worthy of further exploration.

## Conclusion

Focal adhesion plays a crucial role in multiple processes of tumour metastasis. As a bridge between cells and ECs, focal adhesion of tumour cells not only creates a favourable microenvironment for metastasis by promoting the remodelling of the tumour matrix, but also promotes processes such as tumour angiogenesis and matrix degradation by regulating intercellular adhesion and signalling. These mechanisms support the occurrence and development of tumour metastasis.

In addition, the migratory behaviour of tumour cells, including the establishment of cell polarity, the initiation of motility, the assembly of focal adhesions at the leading edge and the dissociation of focal adhesions at the trailing edge, all depend on the dynamic balance of focal adhesions. Metastatic cascade responses of tumours, such as invasion, intravascular penetration and colonisation of distant organs, are also closely related to focal adhesion. Thus, focal adhesion acts as a key regulator at multiple stages of tumour metastasis.

Despite its importance, the assembly and dissociation of focal adhesions and the related molecular mechanisms have not been fully elucidated due to the limitations of research techniques. Especially in tumour cells, the abnormal function of focal adhesion is closely related to the migratory and invasive behaviours of tumour cells, but our understanding of these processes is still not deep enough. Further studies are needed to reveal the dynamics of focal adhesions in tumour cells and how it drives metastatic processes in the tumour microenvironment. These studies are important for our understanding of how dysregulated focal adhesions in tumour cells alters cell behaviour.

In terms of therapeutic strategies, targeting focal adhesion proteins, such as integrins, FAK, and Src kinase, has become a focal point of anti-tumour metastasis research. These targets play a central role in the signalling and mechanosensing of focal adhesions, and inhibition of these proteins can effectively block focal adhesion-mediated tumour metastatic signalling. Currently, several preclinical and clinical trials have been conducted to evaluate the efficacy of these targeted agents, especially in inhibiting tumour angiogenesis, matrix remodelling, and tumour cell migration, showing good potential. In addition to these traditional targets, some additional focal adhesion proteins, such as talin and ILK, are gradually entering the drug development landscape. Compared with FAK and integrins, these proteins show higher specificity in the assembly and regulation of focal adhesions and are thus considered as potential pharmacological inhibitors. Studies have shown that targeting these focal adhesion proteins not only inhibits tumour metastasis, but also reduces the impact on normal cells, making them important in the development of future focal adhesion inhibitors.

However, a major challenge in targeting focal adhesion is how to inhibit tumour cell migration while avoiding adverse effects on the adhesion and migration functions of normal cells. Focal adhesion is widely present in a wide range of cells and is involved in the repair, regeneration and immune regulation of normal tissues, so how to strike a balance between anti-tumour effects and the reduction of adverse effects when designing focal adhesion-targeted drugs is a central issue in current clinical research. If it is possible to target focal adhesion changes specific to tumour cells while minimising interference with healthy cells, this would significantly improve the safety and efficacy of treatment.

Despite the challenges, the study of targeting focal adhesion still offers great potential for tumour therapy. Focal adhesion may not only serve as a future marker for tumour metastasis, but may also become a new therapeutic target to drive the development of precision tumour therapy. With a deeper understanding of focal adhesion function and regulatory mechanisms, future studies are expected to provide new ideas and directions for the development of more efficient and safer anti-tumour metastasis drugs.

## Data Availability

No datasets were generated or analysed during the current study.

## Abbreviations

ECM: Extracellular matrix
ILK: Integrin-linked kinase
HCC: Hepatocellular carcinoma
GF: Growth factor
EGFR: Epidermal growth factor receptor
TME: Tumour microenvironment
ISL: Integrin signalling layer
FTL: Force transducer layer
ARL: Actin regulatory layer
FAK: Focal adhesion kinase
VASP: Vasodilator-stimulated phosphoprotein
SFs: Stressed fibres
IPP: ILK-PINCH-Parvin
Cav-1: Caveolin-1
TCGA: The Cancer Genome Atlas
COSMIC: Catalog of Somatic Mutations in Cancer
GEO: Gene Expression Omnibus
ICGC: International Cancer Genome Consortium
CGGA: Chinese Glioma Genome Atlas
REMBRANDT: Repository for Molecular Brain Neoplasia Data
MNNs: Mutual nearest neighbours
kBET: K- nearest-neighbour batch-effect test
WGCNA: Weighted gene coexpression network analysis
GSEA: Gene set enrichment analysis
GSVA: Gene set variation analysis
PIP2: Phosphatidylinositol 4,5-bisphosphate
PIP5KIγ: Phosphotidylinosotol-4 phosphate 5 kinase γ
PH: Pleckstrin homology
VBSs: Vinculin binding sites
FN: Fibronectin
EGF: Epidermal growth factor
PSM: Plasmonic scattering microscopy
SMT: Single-fluorescent-molecule imaging tracking
PKC: Protein kinase C
YAP: Yes-associated protein
TEAD: TEA structural domain family members
CaMKK: Calcium/calmodulin-dependent protein kinase kinase
MMPs: Matrix metalloproteinases
BM: Basement membrane
CAFs: Cancer-associated fibroblasts
AFM: Atomic force microscopy nanoindentation
SHG: Second harmonic generation
ECs: Endothelial cells
TNFs: Tumour necrosis factors
TGFs: Transforming growth factors
LAP: Latency-associated peptide
PDGF: Platelet-derived growth factor
FGF: Fibroblast growth factor
IGF-1: Insulin like growth factor
PMN: Pre-metastatic niche
BMDCs: Bone marrow-derived cells
EVs: Extracellular vesicles
LN: Laminin
TAM: Tumour-associated macrophage
HSCs: Hepatic stellate cells
CTCs: Circulating tumour cells
CSD: Caveolin scaffolding domain
PROTAC: Proteolysis targeting chimera
SFKs: Src family kinases
CML: Chronic myeloid leukaemia
MTD: Maximal tolerated dose
